# Genome-wide discovery and characterization of flower development related long non-coding RNAs in *Prunus mume*

**DOI:** 10.1186/s12870-019-1672-7

**Published:** 2019-02-11

**Authors:** Xinxin Wu, Ting Shi, Shahid Iqbal, Yong Zhang, Lin Liu, Zhihong Gao

**Affiliations:** 1 College of Horticulture, Nanjing Agricultural University, Nanjing, 210095 China; 2 Jiangsu Key Laboratory for Horticultural Crop Genetic Improvement, Nanjing, 210095 China; 3 Guangdong Provincial Key Laboratory for Plant Epigenetics, College of Life Sciences and Oceanography, Shenzhen University, Shenzhen, 518060 China

**Keywords:** lncRNAs, miR172d, Pistil differentiation, *Prunus mume*, RNA-seq, Hormones

## Abstract

**Background:**

Long non-coding RNAs (lncRNAs) are transcripts more than 200 bp in length do not encode proteins. Up to the present, it has been reported that lncRNAs play an essential role in developmental processes through their regulatory functions. However, their characteristics, expression inheritance patterns, and functions in *Prunus mume* are quite unidentified.

**Results:**

In this present study, we exposed the specific characters of pistil development process between single pistil cv ‘Qingjia No.2’ (QJN2) and multiple pistils cv ‘Da Yu’ (DY). We found that early October is the key stage for pistil differentiation. The similarity epidermis was observed between two types of pistil. We also further investigated a complete pistil development lncRNA profiles through RNA-seq in *Prunus mume*. 2572 unique lncRNAs and 24,648 genes mapped to *Prunus mume* genome, furthermore, 591 novel lncRNAs were predicted. Both unique lncRNAs and novel lncRNAs are shorter in length than the mRNAs, and the overall expression level of lncRNAs was lower than mRNAs in *Prunus mume*. 186 known lncRNAs, 1638 genes and 89 novel lncRNAs were identified as significant differential expressed in QJN2 compared with DY. We predicted 421 target genes of differentially expressed known lncRNAs (DEKLs) and 254 target genes of differentially expressed novel lncRNAs (DENLs). 153 miRNAs were predicted interacted with 100 DEKLs while 112 miRNAs were predicted interacted with 55 DENLs. Further analysis of the DEKLs showed that the lncRNA of *XR_514690.2* down-regulated its target ppe-miR172d, and up-regulated *AP2*, respectively. Meanwhile, the other lncRNA of TCONS_00032517 induced cytokinin negative regulator gene *A-ARR* expression via repressing its target miRNA ppe-miR160a/b in DY. At the same time we found that the A*P2* expression was significantly up-regulated by zeatin (ZT) treatment in flower buds. Our experiments suggest that the two lncRNAs of *XR_514690.2* and TCONS_00032517 might contribute the formation of multiple pistils in *Prunus mume*.

**Conclusion:**

This study shows the first characterization of lncRNAs involved in pistil development and provides new indications to elucidate how lncRNAs and their targets play role in pistil differentiation and flower development in *Prunus mume*.

**Electronic supplementary material:**

The online version of this article (10.1186/s12870-019-1672-7) contains supplementary material, which is available to authorized users.

## Background

The ‘central dogma’ demonstrates genetic information that moves toward the unidirectional side from DNA to mRNA and then towards protein. However, different types of non-coding RNAs (ncRNA) with different sizes and structures have been described making this definitive theory inadequate for some conditions [1]. NcRNAs are mainly produced from the eukaryotic transcriptome. Based on their length, ncRNAs can be divided into small ncRNAs (shorter than 200 bp) and long ncRNAs (lncRNAs) [2]. LncRNAs have been defined as non-protein coding RNAs having more than 200 bp in length [3]. LncRNAs can originate from exonic, intronic, intragenic, intergenic, promoter regions, 3′- and 5′- UTR, enhancer sequences that transcribed in either sense or antisense direction [4]. Unlike protein-coding RNAs and other types of ncRNAs, the functions of lncRNAs are complicated and obscured owing to their sequences or structures. However, a lot of work has been done to identify and functionally analyze lncRNAs in mammals by sequencing, but the function and mechanism of lncRNA remains to be elucidated [5].

In plants, only a few lncRNAs have been identified and functionally studied including *Arabidopsis* [6]*, Zea mays* [7], tomato [8] and *Manihot esculenta* [9]. LncRNAs have universal and strong functions to regulate gene expression at epigenetic, transcriptional and post-transcriptional level. Nevertheless, it has been shown that lncRNAs play a vital regulatory role in plant biological processes, including gene silencing, stress response, flowering time regulation, flower and pollen development [2, 10–12]. *COOLAIR* and *COLDAIR*, two lncRNAs from *Arabidopsis*, act as a floral repressor, have been characterized by *FLOWERING LOCUS C* [13, 14]. According to stress expression profile, 22 lncRNAs were found associated with abiotic stress response in *Arabidopsis* [6]. Through the combined analysis of 17 lncRNAs, 840 mRNAs and known miRNAs from whole-genome, we explored a widespread existence of competitive endogenous RNAs (ceRNAs) mediated by lncRNAs in Maize, and the outcomes showed seven novel lncRNAs as prospective functional ceRNA [7]. Based on RNA-seq datasets from 35 different flower and fruit tissues of diploid strawberry, 5884 lncRNAs were identified from 3862 loci, and their potential effects on fruit and flower development were emphasized [12]. In the case of *LncRNA1459* mutants, ethylene and carotenoid biosynthesis related genes were distinctly down-regulated in tomato [15].

LncRNAs have the potential to sponge microRNA (miRNAs) and regulate the expression of mRNA [16]. Some lncRNAs hold miRNA binding sites, that act as an endogenous target mimics to bind with miRNAs and condense its repression on their targets [17]. In rice, lncRNA osa-eTM160 attenuated the repression of osa-miR160 on *osa-ARF18* mRNAs throughout early anther developmental stages over target mimicry ways that consequently help to regulate seed size and setting [18]. Emerging suggestion indicates that lncRNAs can also guide gene expression through two manners either in *cis-*acting (on neighboring genes) or in *trans-*acting (on distantly located genes). In *Populus*, computational analysis predicted 939 potential *cis*-regulated and 965 potential *trans*-regulated target genes for GA-responsive lncRNAs. These potential target genes take part in various biological processes that influence growth and wood properties [19]. In *Cassava*, *trans*-regulatory network analysis proposed that numerous lncRNAs are associated with secondary metabolites biosynthesis, sucrose metabolism, and hormone signal transduction pathway [9].

Plant hormones are small signaling molecules that are critical to most aspects of plant growth, differentiation, and development. Cytokinin has an essential role in the regulation of plant development and growth. In tobacco, reduce levels of endogenous cytokinin leads to lower meristems activity [20]. In maize, cytokinin can determine the fate of pistil cell during floret development [21]. Cytokinin regulates gene expression, like *AP2* up-regulated in response to cytokinin in *Arabidopsis* [22]. Auxin is long-distance signaling hormone that modulates the growth at the entire plant level. Differences in auxin content control many developmental processes including meristems patterning. Auxin mainly located at the initiation site of floral primordium, and activate the auxin response factor MONOPTEROS/ARF5, which directly activates LEAFY transcription to trigger floral fate [23]. Gibberellins (GAs) involved in controlling the flowering, their regulation in the developmental process and also partially control the expression of the floral homeotic genes [24].

Japanese apricot (*Prunus mume* Sieb. et Zucc.), an essential member of *Rosaceae*, is a fruit and ornamental crop originated in China for more than 7000 years ago [25]. *Prunus mume* is one of the most precious processed fruit with great economic importance used as preserved and in alcoholic beverage industries. Generally, in *Prunus mume*, a perfect flower retains a normal pistil development leads to single fruit formation. However, *Prunus mume* also own a few varieties with two or more pistils, which lead to multiple fruit formations while others sterility result decreases the yield and low fruit quality. In addition, the molecular mechanism of multiple pistil formations is quite unknown. In order to screen out the potential lncRNAs regulating pistil number, we systematically studied and characterized lncRNAs expression analysis in *Prunus mume* flower buds at the genome-wide scale. Furthermore, these studies also help to deepen our understanding of lncRNAs in pistil differentiation and flower development in *Prunus mume*.

## Results

### Identification of key pistil differentiation stages and similarity epidermis in QJN2 and DY

**Fig. 1:**
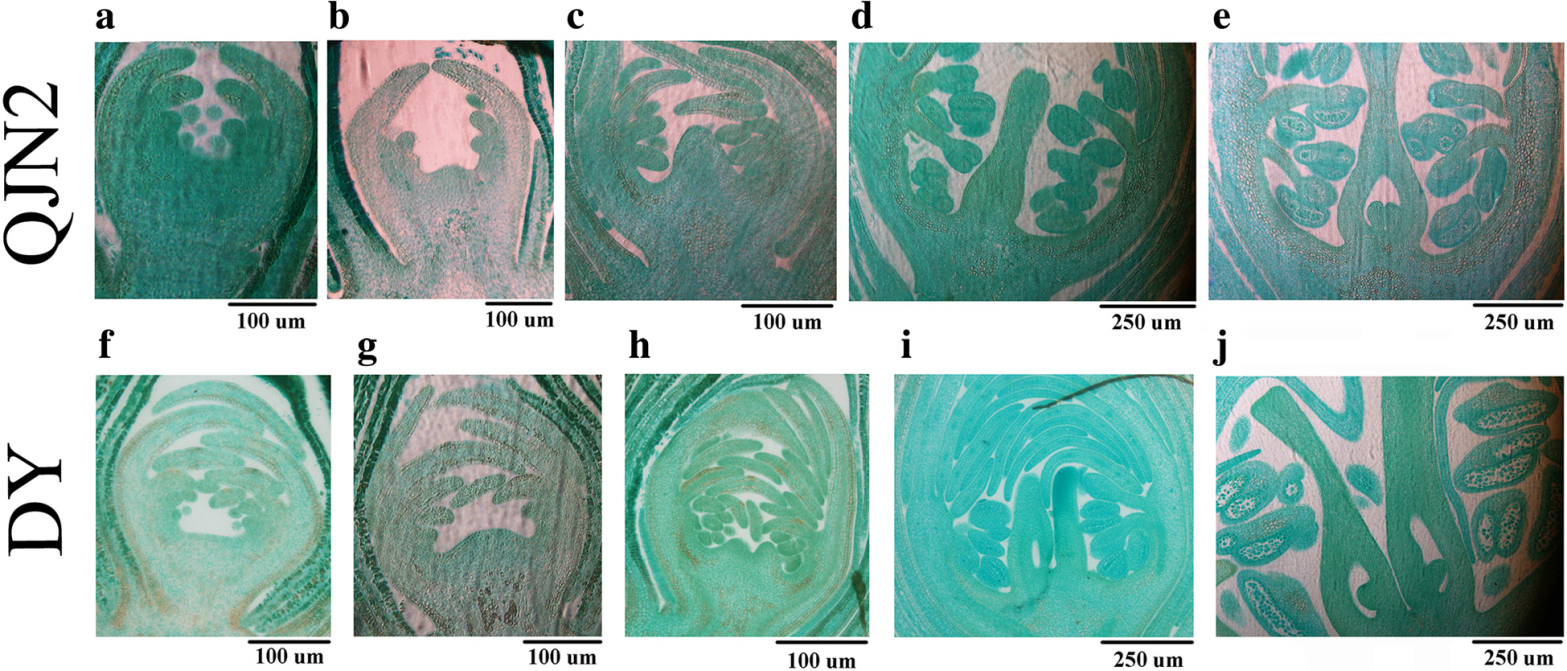
Paraffin section observed differentiation procession of pistil development between *Prunus mume* cultivars QJN2 and DY. **a** and **f** are the pre-differentiation stage of pistil development. **b** and **g** are the early differentiation stage of pistil development. **c**-**d** and **h**-**i** are the differentiation stage of pistil development. **e** and **j** are the late differentiation stage of pistil development. Scale bars are shown in the figure

Based on paraffin section results, the process of pistil differentiation and development of QJN2 and DY was observed (Fig. 1). Late September, the pistil primordial was in pre-differentiation stage (Fig. 1a) and sepals, petals, and stamens have appeared. In early October, the pistil primordial entered in early differentiation stage and started to expand (Fig. 1b). From late October to November, the pistil primordial was in the differentiation stage, and cell division was strong. At this stage, the pistil can also be observed, just like one bulge for QJN2 while two bulges for DY. Moreover, stamens have formation during this phase (Fig. 1c-d). In early December, the pistil primordial entered in the late differentiation stage, and ovules were formed (Fig. 1e). The same pistil differentiation process was observed in the DY cultivar (Fig. 1f-j). Therefore, we found that early October is the key stage for pistil differentiation.

**Fig. 2:**
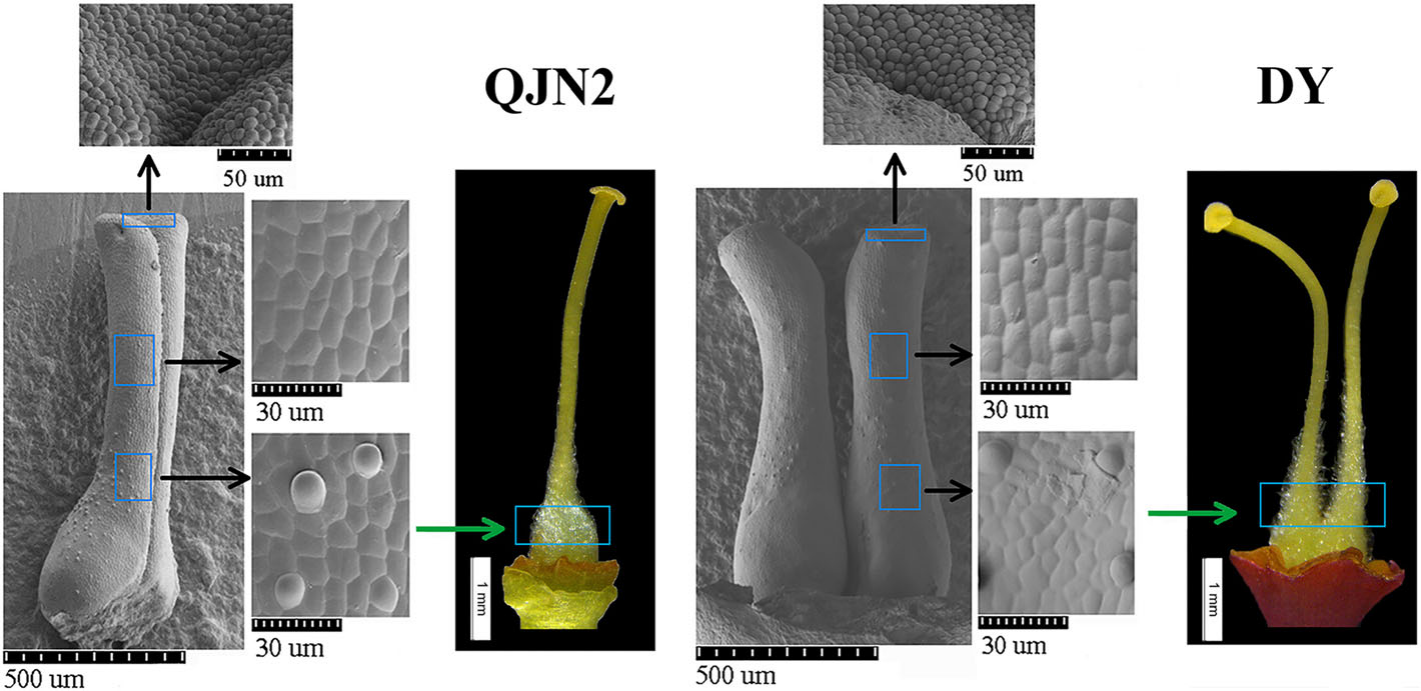
Similar cell shape of pistil surface was observed by SEM photomicrographs in *Prunus mume*. QJN2 is a single pistil while DY is multiple pistils. The scales are shown in the picture

Scanning electron microscopy (SEM) used to obtain the shape of an epidermal cell of the pistil (Fig. 2). By comparing the stigma, middle and base section of the stylus between the QJN2 and DY, we observed similar cell shape between the two type pistils. The stigma owned tight cell, while base section owned stigmatic papillae. The convex-shape cells elongated and formed stigmatic hairs during the later stage.

### Analysis of RNA-seq data, identification and characterization of lncRNAs in *Prunus mume*

To screen out potential lncRNAs involved in regulating pistil number, we performed whole-transcriptome strand-specific RNA sequencing from the flower buds of QJN2 and DY at early differentiation stage, including three biological replicates (QJN2 1, QJN2 2, QJN2 3, DY 1, DY 2, and DY 3). Over 700,000,000 raw sequence reads obtained from six samples with more than 88% clean reads (Table 1). Approximately 70, 72, 73, 80, 77 and 73% of these clean reads were mapped reads and 64, 66, 67, 74, 71 and 67% were unique mapped reads in six libraries, respectively (Additional file 1). These reads evenly distributed across eight chromosomes in *Prunus mume* (Additional file 2a). A set of unique reads was mapped to intergenic regions (24.26%/23.47%/19.94%/9.86%/11.96%/14.44%, QJN2 1/QJN2 2/QJN2 3/DY 1/DY 2/DY 3), exonic (63.84%/65.95%/70.16%/85.68%/81.91%/77.84%, QJN2 1/QJN2 2/QJN2 3/DY 1/DY 2/DY 3) and intronic (11.90%/10.58%/9.90%/4.46%/6.13%/7.72%, QJN2 1/QJN2 2/QJN2 3/DY 1/DY 2/DY 3) as shown in Additional file 2b. Pearson correlation coefficient was used to reflect the degree of correlation between three biological replications. The result showed that three biological replications of per pistil type samples indicated high correlation, as QJN2 with a Pearson’s correlation coefficient ranging from 0.994 to 1 while DY from 0.748 to 0.932 (Additional file 2c).

**Table 1:** Statistical analysis of RNA-Seq reads for two samples with three biological repeats in *Prunus mume*

Samples	Raw reads	Adaptor	Tags containing N	Low reads	Clean Q20	Clean Q30	Total clean reads	Total base	Clean base
QJN2 1	141,145,838 100%	47,712 0.03%	22,104 0.02%	6,435,219 4.56%	98.16%	94.10%	130,341,396 92.35%	21,171,875,700 100%	19,015,769,884 89.82%
QJN2 2	128,163,744 100%	38,212 0.03%	20,262 0.02%	6,490,668 5.06%	98.00%	93.65%	117,374,938 91.58%	19,224,561,600 100%	17,137,520,826 89.14%
QJN2 3	141,878,002 100%	70,877 0.05%	22,413 0.02%	8,245,720 5.81%	97.89%	93.36%	127,996,438 90.22%	21,281,700,300 100%	18,697,953,589 87.86%
DY 1	117,067,752 100%	31,995 0.03%	17,947 0.02%	8,528,818 7.28%	97.39%	91.61%	103,843,854 88.70%	17,560,162,800 100%	15,329,287,829 87.30%
DY 2	97,850,702 100%	24,979 0.03%	15,582 0.02%	6,029,934 6.16%	97.89%	93.40%	87,680,252 89.61%	14,677,605,300 100%	12,921,865,985 88.04%
DY 3	105,778,662 100%	55,748 0.05%	16,686 0.02%	6,128,352 5.79%	97.90%	93.43%	95,474,624 90.26%	15,866,799,300 100%	13,940,388,181 87.86%

**Fig. 3:**
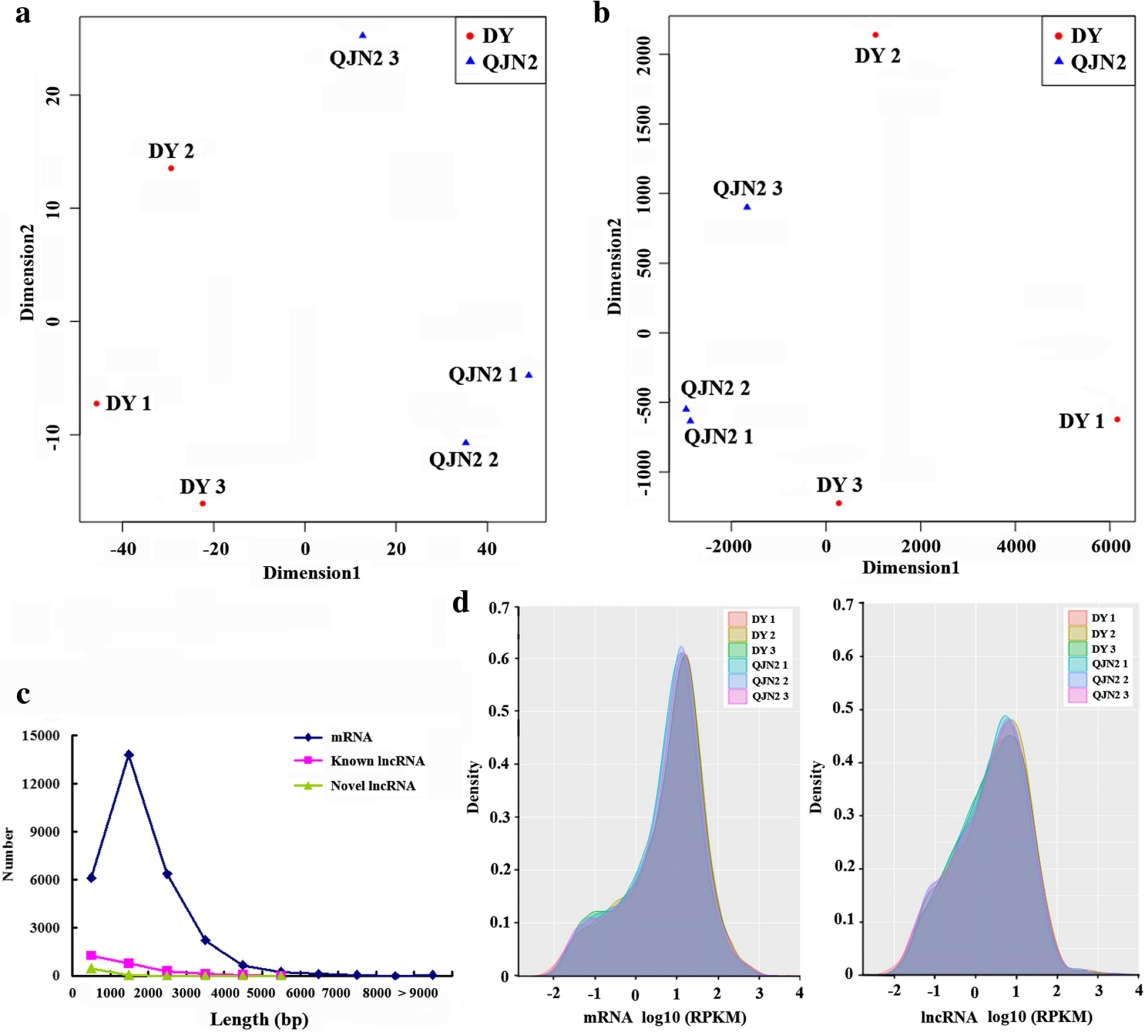
Characteristics of mRNAs, known lncRNAs and novel lncRNAs in *Prunus mume*. a Multidimensional scaling (MDS) plot of mRNA expression differences between the QJN2 and DY. b MDS plot of lncRNA expression differences between the QJN2 and DY. c Length distribution of mRNAs, known lncRNAs and novel lncRNAs. d Expression levels of mRNAs and lncRNAs

All RNA-seq datasets were mapped to *Prunus mume* genome using TopHat. A total of 24,648 known genes and 2572 known lncRNAs were identified. The remaining reads were filtered according to length (transcripts with length less than 200 bp were excluded) and coding potential (coding potential more than 0 were removed) defined as novel lncRNAs. Finally, 591 novel lncRNAs were identified (Additional file 3). Multidimensional scaling (MDS) plot of the mRNA and lncRNA expression showed separation between the QJN2 and DY in dimension1 (Fig. 3a and b). The length of known lncRNAs ranged from 200 to 8360 bp and novel lncRNA ranged from 200 to 5193 bp. The length of known lncRNA was 1314 bp in average and 1751 bp in the median, while known lncRNAs was 682 bp in average and 799 bp in the median. The length of protein-coding mRNAs was 1727 bp in average and 2164 bp in median (Fig. 3c). Therefore, the average length of lncRNAs was shorter than the mRNA. Then, we estimated the expression level of each transcript using reads per kilobase of transcript per million fragments mapped (RPKM) and found that the transcripts in QJN2 and DY were expressed at similar levels. However, the overall expression level of lncRNAs was lower than mRNAs (Fig. 3d). Conservation analysis of novel lncRNAs showed that 352 novel lncRNAs (59.56%) were conserved in other genomes. Most of them were mapped to the *Prunus*, including *Prunus avium*, *Prunus persica*, *Prunus armeniaca*, *Prunus pedunculata* (Additional file 3). The characterizations of lncRNAs in *Prunus mume* were consistent with studies in other plants.

### Identification of differentially expressed genes and lncRNAs in *Prunus mume*

**Fig. 4:**
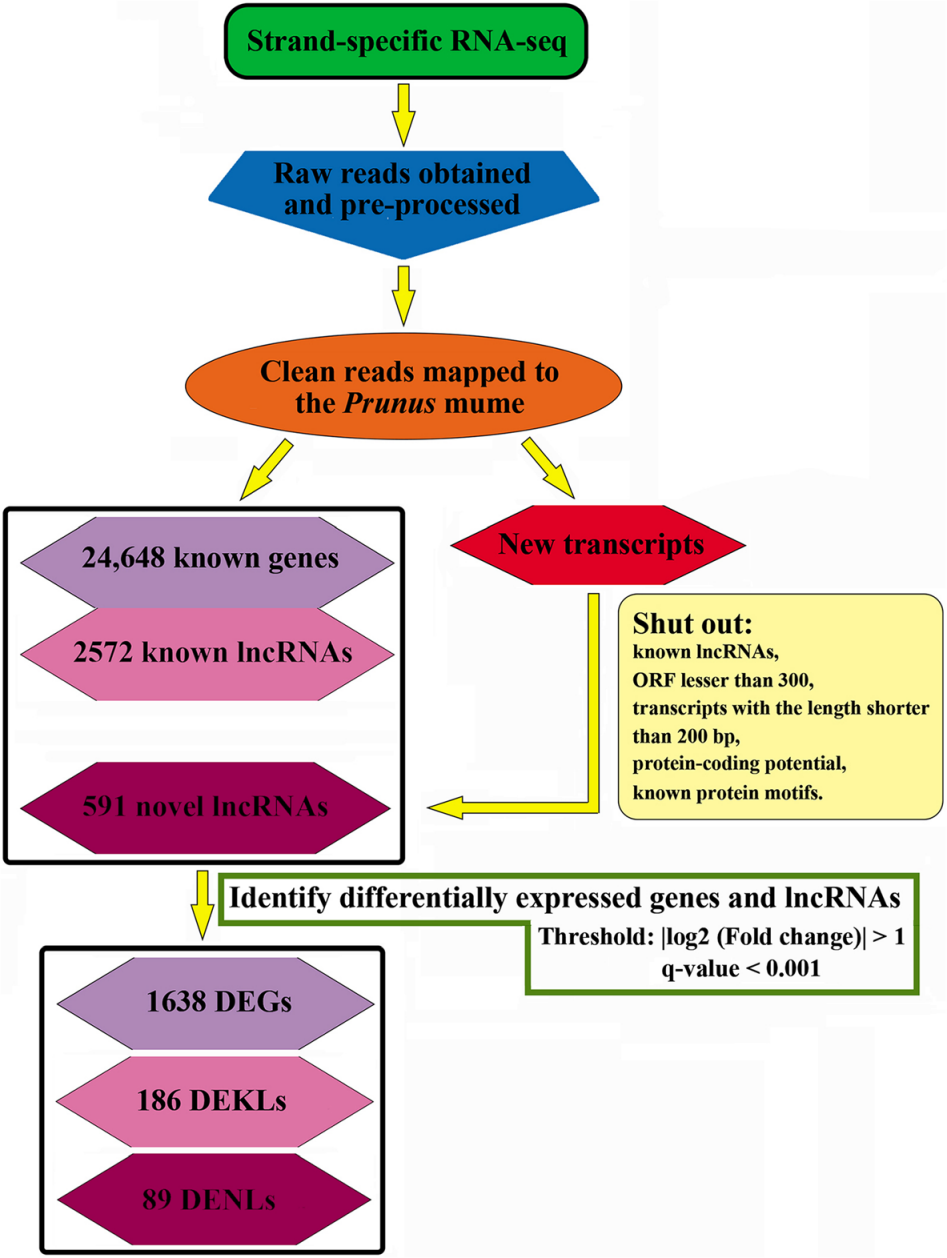
Flow chart on identification of DEGs, DEKLs and DENLs in *Prunus mume*. Single pistil cv QJN2 as control check and multiple pistils cv DY as the treatment group

A total of 24,648 expressed genes were identified and mapped on the *Prunus mume* genome. Contrasts between QJN2 and DY samples showed 1638 significantly differential expression genes (DEGs, the absolute value of log_2_ Fold change > 1 and q-value < 0.001), including 1040 up-regulated and 598 down-regulated genes (Fig. 4, Additional file 4). GO used to evaluate DEGs function classification. The DEGs were categorized into 21 functional groups (*P* value < 0.05 as the threshold), including 10 molecular functions, 4 cellular components and 7 biological processes (Additional file 5a). The pathway analysis results, based on the KEGG database, showed that DEGs be categorized into 27 pathways (Additional file 5b, 6). Metabolic pathways include maximum DEGs (ko01100, 155 genes). Plant hormone signal transduction is the fourth significant enrichment pathway. 18 DEGs on plant hormone signal transduction and 42 DEGs on plant hormone biosynthesis may be the key factors for flower development (Additional file 6).

In this present study, we identified a relatively comprehensive set of *Prunus mume* lncRNAs. Because of RNA-Seq, analysis of assembling, annotation, and filtering of all transcripts was done. A total of 3163 lncRNAs were obtained from all six libraries, including 2572 known lncRNAs (annotated on the *Prunus mume* genome) and 591 novel lncRNAs (new predicated except *Prunus mume* genome). 186 known lncRNAs as significant differential expressed (DEKLs) were identified in buds of QJN2 compared with DY samples, including 15 up-regulated and 171 down-regulated lncRNAs (Fig. 4; Additional file 7). We also found that five lncRNAs were DY-specific expressed lncRNAs. Therefore, the function of lncRNAs needs to be further research.

Expression analysis also showed 89 novel lncRNAs differentially expressed in buds of QJN2 compared with DY, through which 69 were up-regulated and 20 were down-regulated (Fig. 4). Moreover, there were six expressed exclusively in DY flower buds, two expressed exclusively in QJN2 flower buds and 81 co-expressed in both flower buds, respectively (Fig. 4; Additional file 7). Based on the Rfam database (version 12.0), we found 54 novel lncRNAs mapped to 25 families (Additional file 8). Furthermore, we found that these novel lncRNAs were potential as the precursor of miRNA and snoRNA, such as miRNA, MIR390, MIR396, MIR535, MIR156, and snoRNA, snoU36a, snoR44_J54 and snoRD43.

### Flower development related lncRNAs might have function via interacting with miRNAs and genes

**Fig. 5:**
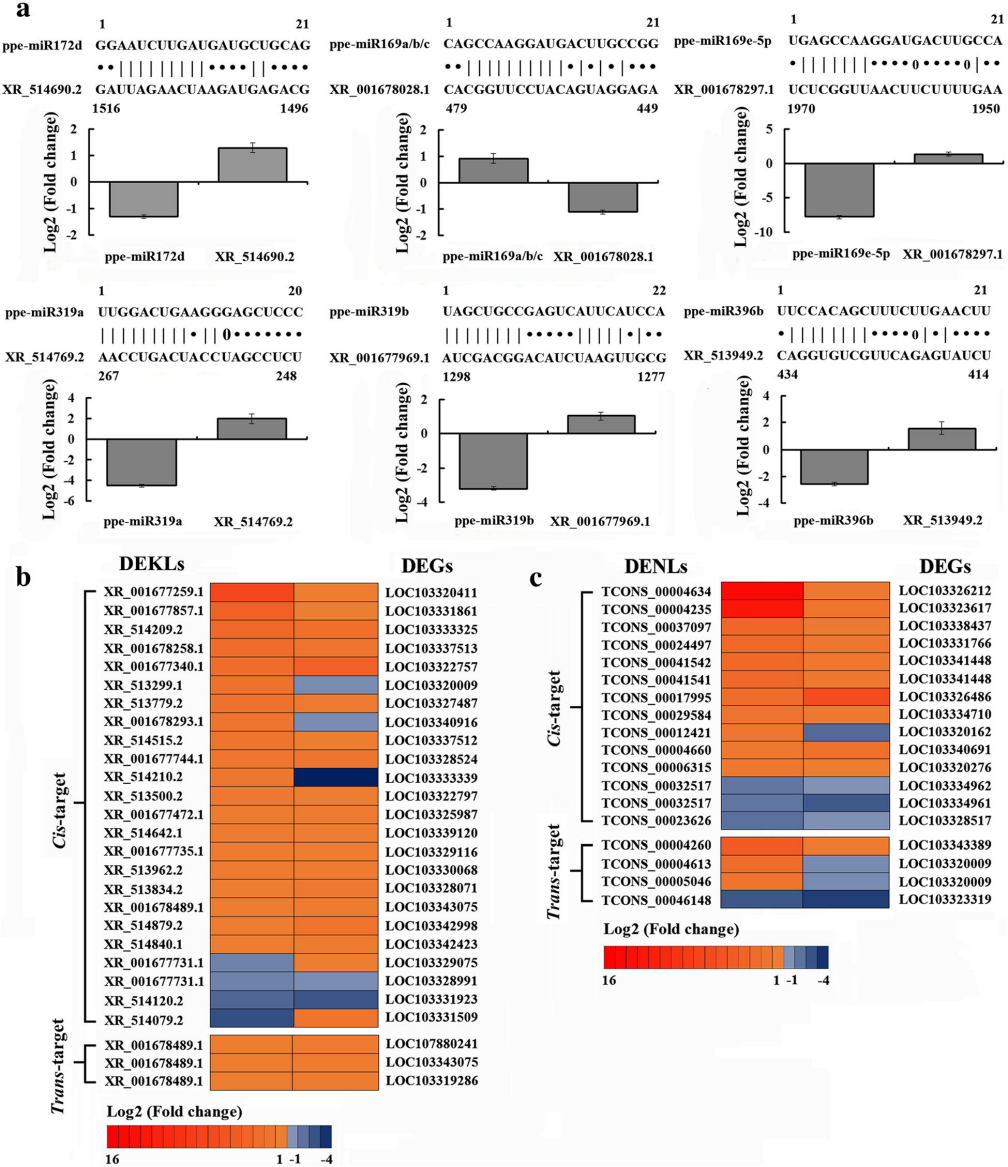
Expression relationship between differentially expressed lncRNAs and their potential targets. a Expression relationship between DEKLs and their potential target miRNAs. Unpaired nucleotides are marked by ‘•’, paired nucleotides are marked by ‘**|**’, G-U pairs are marked by ‘0’. b Expression relationship between DEKLs and their *cis*-target and *trans*-target DEGs. c Expression relationship between DENLs and their *cis*-target and *trans*-target DEGs. Error bars indicate SDs among three biological replicates (*n* = 3)

Three different computational approaches, including MIRANDA, PITA and RNAHYBRID were applied to predict the potential target miRNAs for all DEKLs and DENLs between QJN2 and DY. As a result, only 153 targets miRNAs of 100 DEKLs were predicted by three approaches as shown in Additional file 9. Further analysis revealed that there were 473 miRNA-lncRNA interaction pairs. To explore the relationship between the expression of lncRNAs and their potential target miRNAs, we selected six miRNA-lncRNA pairs and measured their expression through qRT-PCR. A negative relationship among lncRNAs expression and their potential target miRNAs were detected (Fig. 5a).

Furthermore, we predicted DEKLs target genes, divided into 378 *cis*-target genes and 43 *trans*-target genes (Additional file 10). For example, the multiprotein bridging factor 1c (MBF1c) was the *cis*-target gene of *XR_001678293.1* and protein TOPLESS-like (TPL) was the *trans*-target gene of six DEKLs (XR_001677462.1, XR_001678577.1, XR_001678578.1, XR_513648.2, XR_513660.2 and XR_515023.2). Among these potential target genes, 240 *cis*-regulated target genes and 19 *trans*-regulated target genes showed changes in transcript levels (*P* < 0.05). To analyze a relationship between expression of DEKLs and their potential target DEGs expression patterns, we compared their trends of expression between QJN2 and DY. Total of 27 DEKL-DEG pairs were found. Among them, 22 DEKL-DEG pairs (81.48%) showed the same trend, while 5 DEKL-DEG pairs (18.52%) showed an opposite trend. For *cis*-target gene, lncRNA *XR_513962.2*-*XM_016793708.1* (LOC103330068) and *XR_001677259.1*-*XM_008222091.1* (LOC103320411) own the same trend, but *XR_514210.2*-*XM_008236161.2* (LOC103333339) own opposite trend (Fig. 5b). Furthermore, the lncRNA *XR_001677731.1* own both trend, e.g., *XR_001677731.1*-*XM_008231505.1* (LOC103329075) showed same trend while *XR_001677731.1*-*XM_008231419.2* (LOC103328991) showed the opposite trend. For *trans*-target genes, the three DEKL-DEG pairs were shown the same trend (Fig. 5b). Different expression relationships between DEKLs and their potential target genes indicated various regulatory mechanisms of lncRNAs.

We also predicted the target genes and miRNAs interacting with DENLs. A total of 72 DENLs own 221 *cis*-target genes while 19 DENLs own 33 *trans*-target genes (Additional file 10). Among these DENLs, TCONS_00036642 and TCONS_00037216 own the *cis*-target gene AGAMOUS-like MADS-box protein AGL65. To analyze a relationship between expression of DENLs and their potential target DEGs expression patterns, we compared their trends of expression (Fig. 5c). Total of 18 DENL-DEG pairs was found. Among them, 15 DEKL-DEG pairs (83.33%) showed the same trend of expression of potential target gene and lncRNA, while 3 DENL-DEG pairs (16.67%) showed an opposite trend. We predicted the potential target miRNAs for all 89 DENLs between QJN2 and DY. Only 55 DENLs own target miRNAs, which constituted 237 lncRNA-miRNA interactions predicted by three approaches as shown in Additional file 10. The relationship between the DENLs, target miRNAs and genes needs to further research.

Besides the results of lncRNA interacting with miRNAs and genes, we found 16 DEKLs and 8 DENLs only targeting miRNAs while 70 DEKLs and 29 DENLs only targeting genes. This result suggested that most of the lncRNAs own multiple regulatory functions by target miRNAs and genes, but a few of lncRNAs were a single function. Furthermore, a single lncRNA could target several genes or miRNAs.

### The negative relationship among *XR_514690.2*, ppe-miR172d and *AP2*

**Fig. 6:**
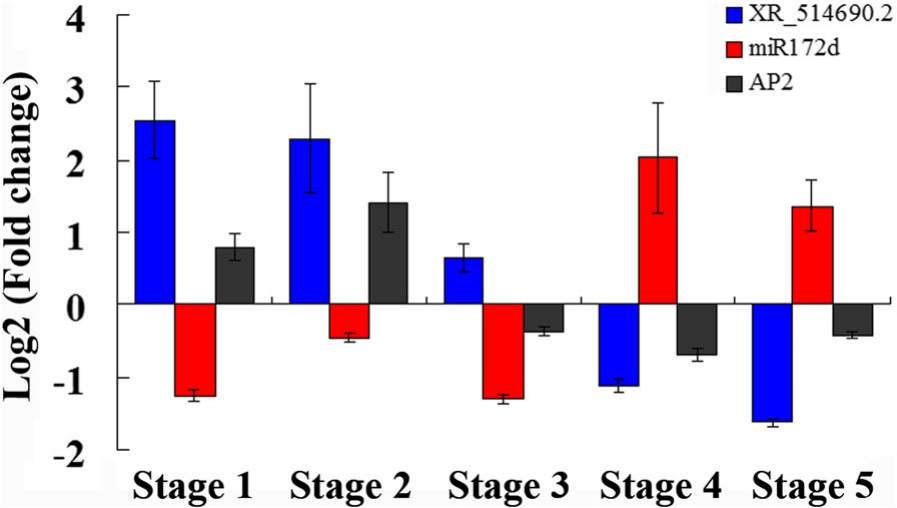
The negative relationship of the gene expressed among *XR_514690.2*, ppe-miR172d and *AP2*. Stage 1 is the pre-differentiation stage. Stage 2 is the early differentiation stage. Stage 3–4 is the differentiation stage. Stage 5 is the late differentiation stage. Error bars indicate SDs among three biological replicates (n = 3)

Based on the results of target miRNAs, we found a lncRNA-miRNA interaction pair *XR_514690.2-*ppe-miR172d. Evidence suggested that miR172 negatively regulate its target gene *AP2* to regulate flower development. According to our gene expression results of *XR_514690.2*, ppe-miR172d and *AP2*, we found a negative relationship between *XR_514690.2* and ppe-miR172d, as well as ppe-miR172d and *AP2* (Fig. 6). The expression of *XR_514690.2* gradually decreased in DY compared with QJN2 during flower development stages. At the pre-differentiation and early differentiation stage, *XR_514690.2* and *AP2* were up-regulated while ppe-miR172d was down-regulated.

### Determination of plant hormone differential content in flower buds of QJN2 and DY

**Fig. 7:**
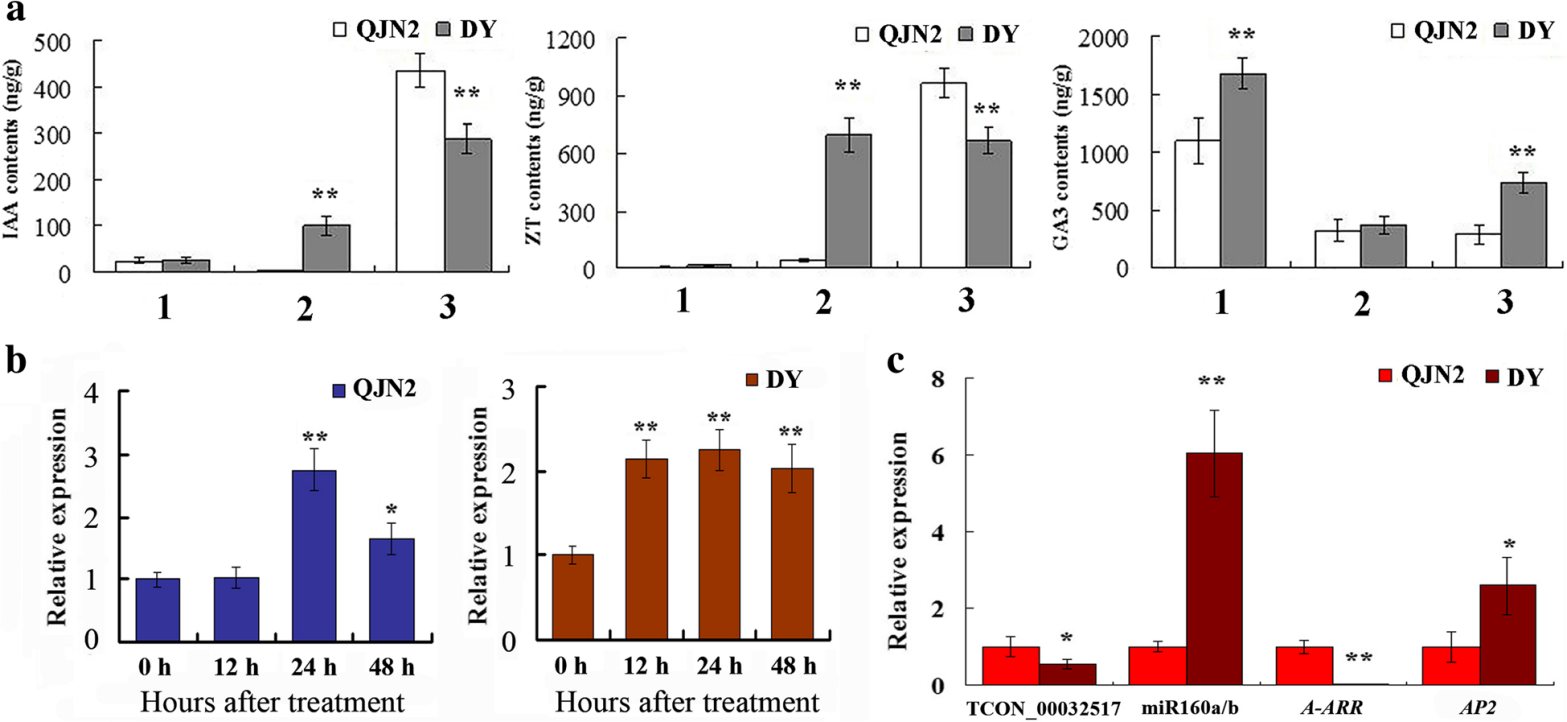
Changes in endogenous plant hormone and *AP2* expression in *Prunus mume* during pistil development. a Changes in endogenous plant hormone in flower buds of *Prunus mume* during pistil development. b *AP2* was up-regulated in response to ZT treatment. c The novel lncRNA TCON_00032517, miRNA miR160a/b, gene *A-ARR* and *AP2* expressed in early differentiation stage. 1: Pre-differentiation stage. 2: Early differentiation stage. 3: Late differentiation stage. Different asterisks (*) indicate significant differences (*, *P* < 0.05; **, *P* < 0.01)

Plant hormones have been reported to play an important role in flower development and its meristems activity. Therefore, we used UPLC to determine the plant hormone content including cytokinin (ZT, zeatin), Auxin (IAA, indole acetic acid) and GA_3_, results shown in Fig. 7a. There were no significant differences in IAA and ZT contents between QJN2 and DY flower buds at the pre-differentiation stage. The contents of IAA in DY flower buds remarkably increased at an early differentiation stage while QJN2 are decreased. At the late differentiation stage, both QJN2 and DY remarkably increased compared with early differentiation stage and QJN2 higher than DY. At the pre-differentiation stage, both QJN2 and DY own the lowest levels of ZT content and then increase at early differentiation stage. The levels of ZT content in DY rise rapidly at the early differentiation stage whereas in QJN2 rise rapidly at the late differentiation stage. Unlike IAA and ZT, GA_3_ content was significantly higher at the pre-differentiation stage than the other two stages. At the pre-differentiation and late differentiation stage, the GA_3_ contents in DY were significantly higher than that of QJN2. Further analysis carried to check the relationship between ZT and *AP2* expression (Fig. 7b). By ZT treatment (1 mmol/L), the *AP2* expression was significantly up-regulated in flower buds after 12 h in DY, while after 24 h in QJN2. At the early differentiation stage of pistil development, the highest ZT levels with the highest *AP2* expressed in DY. Based on the lncRNA-miRNA analysis, we found that novel lncRNA TCON_00032517 target of miR160a/b, and *A-ARR* negatively regulated by ppe-miR160a/b. The down-regulated *A-ARR* negatively regulated the cytokinin signaling would cause flower organs indeterminacy by up-regulated expressed of *AP2*. The gene expression analysis is consistent with the regulation process (Fig. 7c).

### Real-time quantitative RT-PCR analysis

**Fig. 8:**
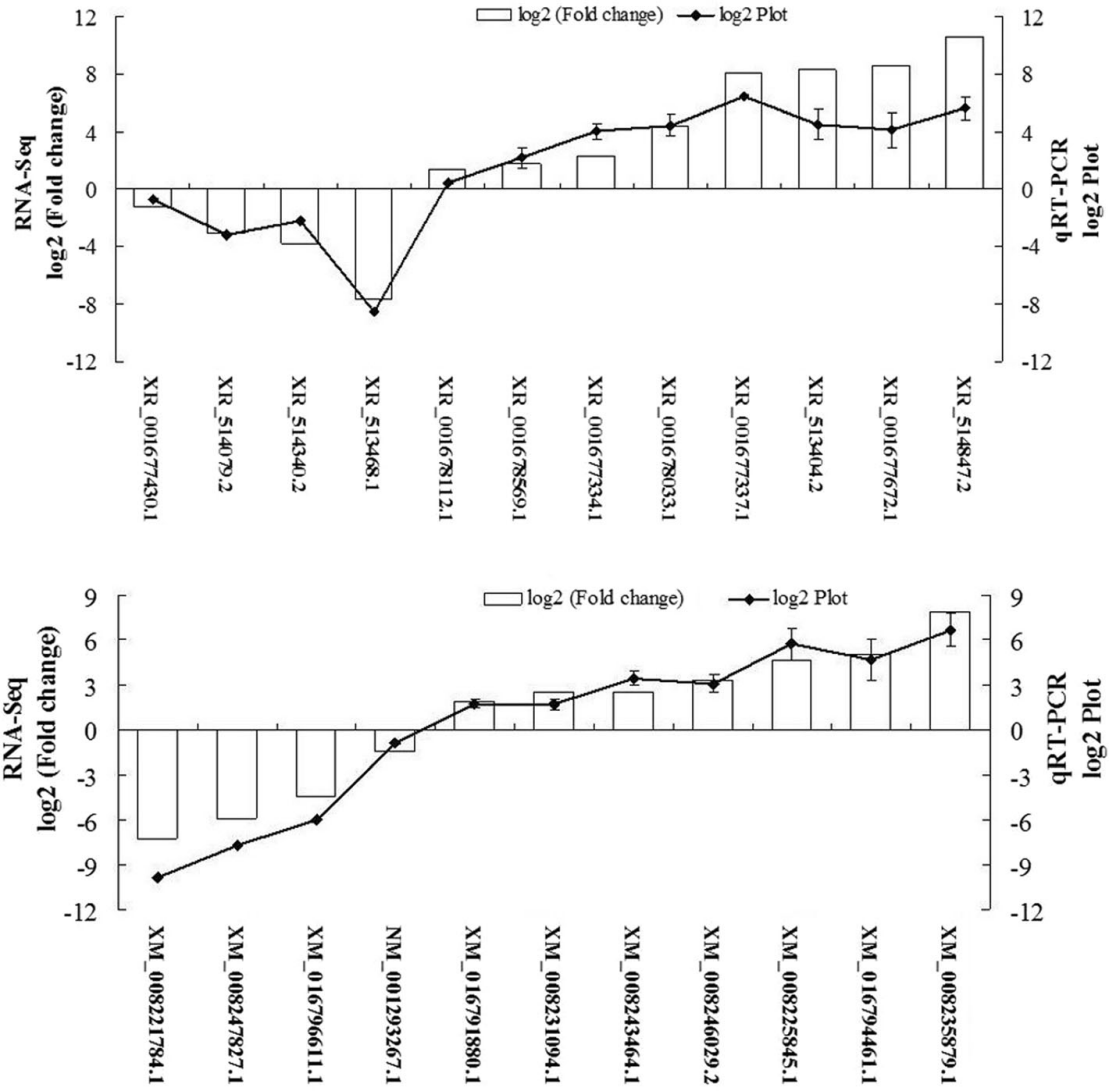
qRT-PCR validation of differential expression genes and lncRNAs in *Prunus mume*

To confirm the consistency of RNA-seq, 11 genes and 12 lncRNAs were randomly selected for qRT-PCR analysis (Fig. 8). The expression of each DEG and DEKL in the different types of pistil samples compared with its abundance from the sequencing data from RNA-seq. The relative expression levels of the genes and lncRNAs were calculated in the qRT-PCR analysis. The result suggested good reproducibility between RNA-seq and qRT-PCR.

## Discussion

### *Prunus mume* lncRNAs features and DEKLs of flower development were identified

The rapid development of transcriptomics sequencing has simplified the identification of thousands of lncRNAs in the plant, but their roles in pistil development remain unknown. To identify differentially expressed genes and lncRNAs, we used comparative transcriptome analysis of single pistil cv QJN2 and multiple pistils cv DY. We identified 24,648 known genes and 2572 known lncRNAs. According to peach deep RNA-Seq results, most of the novel transcribed regions (66.2%) are lncRNAs [26]. Through our stringent lncRNAs identification process, and 591 novel lncRNAs transcripts were identified. However, these lncRNAs identified in our study share similar features with the lncRNAs identified in other plants. Most lncRNAs displayed lower sequence conservation across species than protein-coding genes. Between sorghum and maize, only 25% of lncRNAs were conserved [27]. By genome-wide analysis of lncRNAs in five monocots and five dicot species demonstrated that the lncRNAs displayed high sequence conservation at sub-species and intra-species, but less conserved at inter-species [28]. In our study, conservation analysis of novel lncRNAs showed that 352 novel lncRNAs (59.56%) conserved in other genomes, including *Prunus* (53.30%) and inter-species (6.26%). The results suggested that the novel lncRNAs of *Prunus mume* were higher conservation at intra-species than inter-species. Because of sequence conservation and homology act as indicators of biological function, therefore, understand the function of less conserved lncRNA will be challenging. In *Populus*, the average length of lncRNAs was half that of protein-coding transcripts, and the lncRNAs expression on average at a 5-fold lower level than mRNA [19]. Based on RNA-seq of 80 individuals of *Miscanthus lutarioriparius*, lncRNAs were also significantly shorter than mRNAs in length [29]. Our study showed that the length of known lncRNAs with an average length of 1314 bp while the novel lncRNAs with an average length of 683 bp. Compared with these lncRNAs, mRNAs with an average length of 1728 bp. Therefore, both known lncRNAs and novel lncRNAs in length are shorter than the protein-coding transcripts. Moreover, these lncRNAs were expressed at lower levels than mRNAs in *Prunus mume*. However, the expression of lncRNAs showed high cell or tissue type-specific expression in plant [27]. Moreover, lncRNAs can regulate gene expression through a variety of mechanisms. They can act as a miRNA decoy which activates gene expression through sequestering miRNAs [30]. Similar to lncRNAs in rice, cotton and *Arabidopsis* [16, 31], a proportion of *Prunus mume* lncRNAs were predicted to be miRNAs target. Moreover, lncRNAs can regulatory target genes by *cis*- and *trans*-acting. Therefore, our results implied an important functional pattern relationship among lncRNAs, target miRNAs and target genes. Therefore, our results provide rich information and used for further research.

Based on expression analysis of *Prunus mume* lncRNAs results, we identified 186 DEKLs (7.23%) and 89 DENLs (15.06%) in DY samples compared with QJN2 samples, which suggested the important roles of lncRNAs in regulated flower development. Among these DEKLs, we found that *RLP12* was up-regulated in DY. RLPs are present in many plant species and are concerned to plant growth and development along with resistant to disease. Two *AtRLPs* (*AtRLP2* and *AtRLP12*), share maximum sequence resemblance with *CLV2*, were found to be capable of rescue the *clv2* mutant phenotype as expressed under control of *CLV2* promoter. Loss-of-function of any *CLVs* causes the advanced accumulation of homogeneous stem cells, resultant an enlarged meristems, increased floral organ numbers and altered phyllotaxy [32]. In shoot and floral meristems, the *WUS* gene is mandatory for stem cell identification, whereas the *CLAVATA* (*CLV*) *1*, *2* and *3* genes stimulate organ initiation. The *CLV* genes repress *WUS* at the transcript level and that *WUS* expression is adequate to induce meristems cell identity [33]. Myosins are eukaryotic molecular motors moving beside actin filaments. Current information proposes roles of higher plant myosin at cytoplasmic streaming, cell growth, and plant stature [34]. In the meristems of root tips, cell division hindered, and that cell plates miss-located. AMS, a basic loop-helix-loop (bHLH) tapetum-specific transcriptional factor, has been shown to affect genes expression involved in the transportation of lipids, flavonol accumulation, methyl modification, and pollen wall formation in higher plant [35]. Cell wall degradation associated with an increase in activity of several hydrolytic enzymes like polygalacturonase. Cell wall degradation has observed to take place throughout development contributing to such processes as elongation growth and pollen tube growth. *SWEET* genes, newly identified plant gene family that play a crucial role in pollen development [36].

### Flower development-related lncRNAs form regulative networks with miRNAs and mRNAs

LncRNAs might regulate gene expression either in *cis*- or *trans*-acting [37]. *Cis*-acting lncRNAs have been reported to control the gene expression that is positioned in the vicinity of their transcription sites [38]. *Trans*-acting lncRNAs regulate gene expression at independent loci [9]. In *Arabidopsis*, the lncRNA *COOLAIR* and *COLDAIR* have been reported to repress the expression of *FLOWERING LOCUS C* to affect the flowering [39]. In this study, we found 378 *cis*-target genes and 43 *trans*-target genes by analysis of DEKLs between QJN2 and DY. Based on the expression of potential target gene and lncRNA, a total of 27 DEKL-DEG pairs was found. Among them, 22 DEKL-DEG pairs (81.48%) showed the same trend, while 5 DEKL-DEG pairs (18.52%) showed an opposite trend. The different expression relationships between DEKLs and their potential target genes indicated various regulatory mechanisms of lncRNAs. The function of most lncRNAs is unknown, and the potential target genes of lncRNAs need to confirm experimentally.

During analysis of lncRNA potential target genes, we found the MBF1c was the *cis*-target gene of *XR_001678293.1*. MBF1c is an extremely conserved transcriptional co-activator regulated by several processes, like shoot endothelial cell differentiation, hormone-regulated lipid metabolism, biotic and abiotic stress [40]. 25 DEKLs own *trans*-target genes, consist of 43 lncRNA-gene relationships. Among these, we found 6 DEKLs *trans*-target TPL, up-regulated in DY, demonstrating that AP2 functions as a transcriptional repressor originate from fusions of TPL to DNA-binding domain of AP2. These TPL-AP2 fusions rescue floral defects of *ap2–2* mutants [41]. AP2 binds directly to the large regulatory second intron of *AG* and recruits both *TPL* and the histone deacetylase HDA19, likely explaining the repression of *AG* by *AP2* [42]. We also predicted target genes interacting with DENLs. 72 DENLs own 221 *cis*-target genes while 19 DENLs own 33 *trans*-target genes. Among these DENLs, *TCONS_00036642* and *TCONS_00037216* own the *cis*-target gene AGAMOUS-like MADS-box protein AGL65. AGL65 is necessary for pollen development in plant [43].

New findings proposed that lncRNAs could potentially interact with other classes of non-coding RNAs including miRNAs and modulate their regulatory role through interactions [44]. The miRNAs are important factors in plant development usually negatively regulate gene expression by mediating the cleavage of target mRNAs or by repressing their translation. In our study, we obtain 710 lncRNA-miRNA interactions, including 473 DEKL-miRNA interactions and 237 DENL-miRNA interactions. A few reports contributed to signifying the importance of the microRNA (e.g. miR172 family) in control of flower meristems termination. miR172 regulates stem cell fate and describes the inner boundary of *PISTILLATA* and *APETALA3* expression domain in *Arabidopsis* floral meristems [45]. In *Prunus mume*, miR172 negatively regulated its target gene, like *AP2* [46]. Mutants with reduced miR172 levels, as well as a mutant for *mir172d-1*, exhibit a potential for indeterminacy in several genetic backgrounds. miR172 promotes stem cell termination by down-regulating its target *AP2*, which in turn represses *AG*. Overexpression of *AP2* (*35S:AP2*) in a wild-type resultant solitary subtle phenotypic defects while over-expression of the miR172-resistant version of AP2 (*35S*:*AP2m1/3*) results to entire loss of determinacy through completely indeterminate meristems at the center of the flower [47]. In our study, we found three lncRNAs, *XR_513294.1* (uncharacterized LOC103319808, 1.65), *XR_514692.2* (UPF0481 protein At3g47200-like, 1.36) and *XR_514690.2* (UPF0481 protein At3g47200-like, 1.38) all were up-regulated in DY, targeting the miR172. Through expression analysis of ppe-miR172d, lncRNA *XR_514690.2* and *AP2*, we found that ppe-miR172d and lncRNA *XR_514690.2* was the negative relationship, while ppe-miR172d and *AP2* also negative regulation. The higher level of lncRNA *XR_514690.2* might cause lower levels of miR172 and a higher level of *AP2*, which may result in low *AG* gene expression, and then high *WUS* expression may cause indeterminate flowers in DY.

The miR169 family may have a conflicting effect to that of miR172 on flower determinacy [48]. The miR169 family reduces the expression of *AG* orthologs in petunia and snapdragon, might have a same role in *Arabidopsis* [49]. Decrease in miR169 levels consequently counterbalance the reduction in miR172 in *hen1* and *dcl1/caf* and account for their moderate indeterminacy phenotype. In this study, we predicted 15 DEKLs and 4 DENLs interact with miR169, including *XR_513468.1* (beta-glucosidase 12-like, − 7.63), *XR_001678028.1* (uncharacterized LOC107881413, − 2.35), *XR_514358.2* (uncharacterized LOC103335841, 3.73),, *XR_514769.2* (heat shock 70 kDa protein-like, 1.60), *XR_514692.2* (UPF0481 protein At3g47200-like, 1.36), *XR_514708.1* (60S ribosomal protein L18a-like, 1.22), *XR_514690.2* (UPF0481 protein At3g47200-like, 1.38), *XR_001678297.1* (myosin-11, 1.06), TCONS_00004613 (3.14), *TCONS_00043298* (2.75), *TCONS_00023005* (2.23), *TCONS_00005046* (2.10). From above results, we detected that ppe-miR169a/b/c and ppe-miR169e-5p was negative relationship between QJN2 and DY.

The miRNA319 is critical for flowering, cell growth and development in plants. The role of miR319a and its target gene *TCP* is regulation during pistil development in *Prunus mume* [50]. We found an lncRNA *XR_514769.2* (heat shock 70 kDa protein-like, 1.60) and *XR_001677969.1* (probable serine/threonine protein kinase IRE, 1.18) in DEKL. In addition, the ppe-miR319a and b both down-regulated in DY compared with QJN2. Therefore, pistil-related genes through negative interaction with miR319a might facilitate lncRNAs *XR_514769.2* and *XR_001677969.1*. The *35S:MIR396a* plants showed bent, unfused carpels or single-carpel pistil [51]. In our results, we found lncRNA *XR_513949.2* (uncharacterized protein At4g28440-like, 3.67) was up-regulated in DY. *XR_513949.2* target with ppe-miR396a/b and ppe-miR396b was down-regulated in DY compared with QJN2.

### Roles of plant hormones in flower development

Plant hormones are small signaling molecules that are crucial to most aspects of plant growth, differentiation and development. In the KEGG pathway analysis, plant hormone signal transduction is the fourth significant enrichment pathway. There are 18 DEGs on plant hormone signal transduction and 42 DEGs on plant hormone biosynthesis, including auxins, GAs, cytokinins. Our results also indicated that lncRNAs were associated with plant hormone and regulated flower development.

Plant cytokinin plays an essential role in the regulation of plant development and growth. In tobacco, reduce levels of endogenous cytokinin leads to lower meristems activity [20]. In transgenic *Arabidopsis*, over-expression of *AtCKX3* reduced the number of flowers because of the cytokinin breakdown the decreased rate of primordial formation in flower meristems [52]. A multistep phosphorelay pathway in plants is mediated cytokinin signaling. The type-A *Arabidopsis* Response Regulators (A-ARRs) are primary cytokinin response genes mainly negative regulators of Cytokinin signaling [53]. Cytokinin regulates gene expression has been extensively studied, including *AP2* up-regulated in response to cytokinin [22]. In our study, down-expression of *A-ARR (two-component response regulator ORR9-like)* would increase signaling function in DY. By UPLC results, ZT was higher in DY than QJN2 pistil at the early differentiation stage. The gene expression of *AP*2 showed up-regulated response to ZT treatment. The lower ZT content would lead to lower flower meristems activity and lesser flower organs in QJN2. Incidentally, the *A-ARR* was inhibited in *Pro35S:miR160c* in *Arabidopsis thaliana* [54]. We found a down-regulated DENLs TCONS_00032517 target with ppe-miR160a/b, and *A-ARR* was down-regulated in DY. Our results suggest that lncRNAs would regulate pistil development through influence ZT content and signaling transduction by target miRNAs.

Auxin is long-distance signaling modulates growth at the entire plant level. The difference in auxin changes in local auxin content control many developmental processes, including meristems patterning. Auxin mutants shown floral organ identify defects [55]. In our study, the endogenous hormone content of IAA was higher in DY than QJN2 pistil at early differentiation stage. Furthermore, it has been reported that miR160 inhibits the expression of auxin transcription factors (ARFs) [54, 56]. The down-regulated expression of these lncRNAs target with ppe-miR160a/b would inhibit the auxin functions by down-regulation *ARF* genes in DY, which cause the floral organ indeterminacy in DY.

GAs involved in control flowering and regulation of flower development and partially control the expression of the floral homeotic genes in plants [24]. In *Brassica rapa*, a total of 300 differentially expressed genes and 254 differentially expressed lncRNAs were identified by a comparative transcriptome analysis between control and vernalized samples. Through co-localization networks analysis of differentially expressed genes and lncRNAs, the correlated genes were mapped to the plant hormone signal transduction pathway and increased GA_3_ content [57]. Furthermore, genome-wide identification of GA-responsive lncRNAs has done in Populus, and 410 lncRNAs shown gene expressed changes in response to GAs [19]. In our results, the increased content of GA and up-regulated scarecrow-like protein 28 would promote GA signal, which assists in cell division in floral meristems in DY.

## Conclusions

**Fig. 9:**
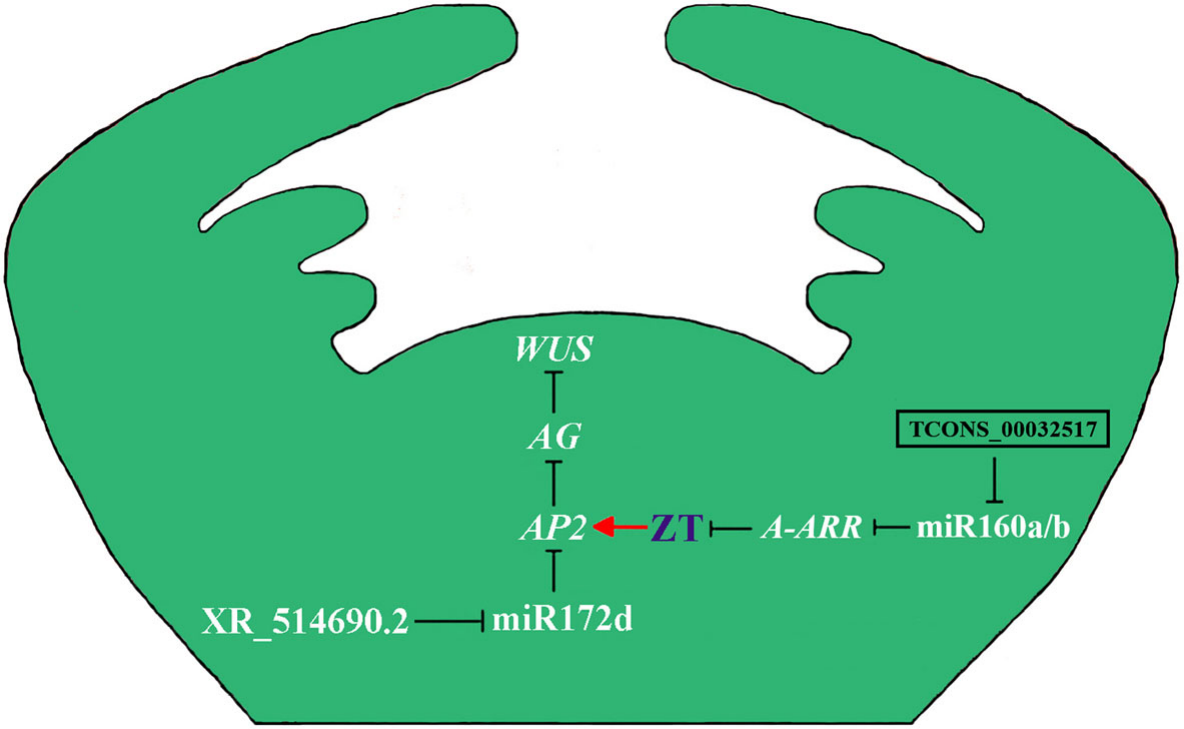
Model of multiple pistil formations in *Prunus mume*

In this present study, we defined a comprehensive pistil development lncRNA profiles in *Prunus mume*. We further revealed conserved and specific characters of pistil development process between two carpel types. 186 known lncRNAs and 89 novel lncRNAs were identified as significant differential expressed in QJN2 compared with DY. We predicted 421 target genes of DEKLs and 254 target genes of DENLs. 153 miRNAs were predicted interacted with 100 DEKLs while 112 miRNAs were predicted interacted with 55 DENLs. Further analysis of the DEKLs showed that the lncRNA of *XR_514690.2* down-regulated its target ppe-miR172d, and up-regulated *AP2*, respectively. Meanwhile, the other lncRNA of TCONS_00032517 induced cytokinin negative regulator gene *A-ARR* expression via repressing its target miRNA ppe-miR160a/b in DY. At the same time we found that the A*P2* expression was significantly up-regulated by ZT treatment in flower buds. Our experiments suggest that the two lncRNAs of *XR_514690.2* and TCONS_00032517 might contribute the formation of multiple pistils in *Prunus mume*. (Fig. 9).

## Methods

### Plant materials collection

In this study, *Prunus mume* cv ‘Qingjia No.2’ (QJN2, single pistil) and ‘Da Yu’ (DY, multiple pistils) grown at the National Field GenBank for Japanese apricot located in Nanjing, Jiangsu Province, China used for these experiments. Samples were collected from 20 September to 27 December per week for different assays. All the collected flower buds were instantly frozen in liquid nitrogen and then stored at − 80 °C for further use.

### Paraffin section

To understand the pistil development and differentiation process of *Prunus mume*, the longitudinal section of flower bud from two cultivars QJN2 and DY at different stages was observed by the paraffin method [58]. Samples were collected from 20 September to 27 December with a one-week interval. The collected flower buds immediately fixed by FAA for paraffin section.

### SEM of pistil epidermal cell morphology in *Prunus mume*

SEM was performed to perceive the variation in surface structures of different types of pistils. Late differentiation stage samples of 20 December were prepared by depositing a drop of diluted suspension in ethanol on a silicon wafer and then examined at 1.0 kV under SEM (Hitachi SU8010, Japan) at various magnifications.

### RNA extraction, library construction, and sequencing

Based on the paraffin section results, early differentiation stage buds (an important stage for pistil formation) were used for RNA extraction and library construction. Each collected was performed with three biological replicates for high-throughput RNA-sequencing, respectively. Total RNA was isolated from flower buds at the early differentiation stage using Trizol reagent (Life Technologies, Carlsbad, CA, USA) followed the manufacturer’s protocols. Additional on-column Dnase digestions were performed during the RNA purification using an RNase-Free Dnase Set (Qiagen, Valencia, CA, USA). The RNA concentration was detected by NanoDrop ND-1000 spectrophotometry (NanoDrop Technologies, Rockland, DE, USA) and Agilent 2200 TapeStation (Agilent Technologies, USA) measured the quality control. Strand-specific RNA-Seq libraries were built using qualified RNA according to RNA Sample Preparation Guide. Subsequently, the library construction and Illumina sequencing were followed the standard procedures performed at Guangzhou RiboBio Co., Ltd. Stranded libraries solitary removing rRNAs were constructed using Epicentre Ribo-Zero rRNA Removal Kit (Illumina, San Diego, CA, USA). The strand-specific libraries were sequenced on an Illumina HiSeq 3000 instrument that generated 150 bp paired-end reads. The libraries named as QJN2 1, QJN2 2, QJN2 3, DY 1, DY 2, and DY 3, respectively. The sequencing data have been submitted to the NCBI Sequence Read Archive (SRA accession number: QJN2 1: SRR7007150, QJN2 2: SRR7027384, QJN2 3: SRR7012247, DY 1: SRR7006118, DY 2: SRR7006119, DY 3: SRR7006910).

### Read mapping and transcriptome assembling

After RNA-Seq, raw reads obtained and pre-processed. Adapters, shorter and low-quality reads were removed and read with a number of N bases accounted for more than 10% were trimmed. After that, all the clean reads were mapped to the *Prunus mume* genome (https://www.ncbi.nlm.nih.gov/genome/?term=prunus%20mume) using TopHat (v2.0.12) with two reads allowed mismatches and two distinct but nearby exons merged into a single exon [59]. Cufflink was used to assemble the mapped reads. ANNOVAR software was used to analyze and annotate the mapped reads.

### Unique lncRNA identification and novel lncRNA predicted

Based on the mapped results, we identified known lncRNAs as unique lncRNAs from the *Prunus mume* genome, while novel lncRNAs predicted from transcripts of transcriptome assemblies according to the characteristic of lncRNA [31]. The flow chart on the lncRNA identification pipeline has shown as Fig. 4. Novel lncRNAs were shut out transcripts with the length shorter than 200 bp, ORF lesser than 300, exon count lesser than 2, the known protein-coding transcript and known lncRNAs. The ORF analysis was performed using BioPerl (version 1.6.923) to find all possible ORF. Transcripts were aligned in the Pfam and Swiss-Prot databases to remove protein-coding domains and encoding proteins. Their non-coding potentials were predicted using Coding-Non-Coding Index (CNCI, version 2) and Coding Potential Calculator (CPC, version 0.9-r2) in combination. A cut off score of less than − 1 was used to select for transcripts with non-coding potential for CPC, whereas for CNCI we chose transcripts that had a negative non-coding prediction. In this study, Rfam database (version 12.0) [60] and blast software (version 2.2.25) used for family analysis of the novel lncRNAs. All the novel lncRNAs sequences predicted in our study were blast with a cut-off E-value <1e-10 and > 20% sequence identity to other genomes were defined as conversed lncRNAs.

### Analysis of DEGs, DEKLs and DENLs

Genes and lncRNAs expression level was measured with normalized counts of reads by their respective length using Cufflinks. RPKM was applied to signify the normalized expression value. A rigorous algorithm was used to identify differentially expressed genes (DEGs), differentially expressed known lncRNAs (DEKLs) and differentially expressed novel lncRNAs (DENLs) between the different samples [61]. Genes and lncRNAs were supposed to significant differential expression with q-value < 0.001, a false discovery rate (FDR) < 0.01, and a relative change in threshold of two-fold in sequence counts across the libraries. The DEGs were performed by GO and pathway enrichment analysis. The GO enrichment analysis was using Blast2GO. The enrichment pathway analysis was done using the KEGG (Kyoto encyclopedia of genes and genomes) database.

### Target gene and miRNAs prediction of DEKLs and DENLs

The prospective target genes of DEKLs and DENLs were predicted according to their regulatory effects, including *cis*- and *trans*-target. The algorithm searches for potential *cis*-target genes are substantially near to lncRNA (within a 10 kb window upstream or downstream) by using genome browser and genome annotation. The algorithm, searches for potential *trans*-targets in mRNA database, based on mRNA sequence complementarity and RNA duplex energy prediction, assessing the influence of lncRNA binding on whole mRNA molecules. Firstly, BLAST used to choose target sequences corresponding to the lncRNA (E-value <1e-5 and identity ≥95%). Then, RNAplex (G < − 20) software used to analyse the corresponding energy between two sequences for additional screening and select potential *trans*-acting target genes. In order to predict the function of differently expressed lncRNAs, target genes were analyzed by GO enrichment and GO terms with Q < 0.05 were considered to be significantly enriched.

Three different computational approaches, including MIRANDA, PITA and RNAHYBRID were used to identify DEKLs and DENLs potential target miRNAs between DY and QJN2. Only targets identified by all three approaches were considered further.

### miRNA isolated and expression analysis by qRT-PCR

The same samples, as RNA-seq, were used to isolate low-molecular-weight RNAs according to Wang et al. [62]. Stem-loop qRT-PCR method was used to detect miRNAs, and all primers showed in Additional file 11. qRT-PCR performed as described earlier. The reactions were incubated at 95 °C for 3 min, followed by 40 cycles at 94 °C for 20 s, 62 °C for 20 s and 72 °C for 40 s. The 5S used as an internal control for an individual sample. For each biological replicate, three technical replicates were performed.

### Plant hormone content determination using UPLC

By the RNA-seq results, we found that the plant hormone signal transduction is the fourth significant enrichment pathway. Plant hormones have been reported an important role in flower development and its meristems activity. Therefore, we selected candidate hormones, including GA_3_, ZT, and IAA, and detected their levels in flower buds of QJN2 and DY at the pre-differentiation stage, early differentiation stage, and late differentiation stage. Extraction, purification and quantitative analysis of the hormones performed according to the methods described by Chen and Yang [63] using an Ultra Performance Liquid Chromatography (UPLC) with a slight modification. 1 g flower buds frozen in liquid nitrogen were ground to fine powder and then immersed in 5 ml 80% precooled methanol (4:1, *v*/v, − 20 °C). After that, 5 ml precooled methanol was used to rinse the mortar and pestle and collected the mixture in 50 ml tube. The mixture extracted under dark room at 4 °C for 12 h and then centrifuged at 4 °C for 15 min at 10000 rpm. The supernatant collected in a new 50 ml tube and sediment immersed in 5 ml 80% precooled methanol again by the described earlier. Combine the supernatant and add 0.2 g crosslinking polyvinylpyrrolidone to shock for 1 h under dark room at 4 °C. After that, the mixture was centrifuged for 15 min at 10000 rpm under 4 °C. The supernatant was collected and filtered by Sep-Pak C18 column to reduce impurities. The filtrate was freeze-drying for more than 36 h to get powder. Then, used 1 ml precooled methanol solvate the powder. Filtration was done of each supernatant solution using 0.45 Millipore filter (organic system), and stored at − 20 °C until quantification of plant hormone by UPLC.

UPLC (ACQUITY UPLC H-Class Core System, Waters, Inc., USA) was equipped with a T3 column (3 μm particle sizes, 100 mm × 2.1 mm, Waters, USA) for separation. The mobile phase was water: acetic acid (99.5:0.5) as solvent A, and methanol as solvent C. The flow rate was 0.35 ml/min and the column temperature set at 30 °C. The injection volume was 2 μl in the Waters system. To obtain chromatograms, hormones perceived by UV absorbance at 256 nm. Under these conditions, the retention times for plant hormones were ZT 3.6 min, GA_3_ 5.3 min, and IAA 6.1 min. Each sample inoculated three times for biological replication.

### ZT treatment effect the AP2 gene expression

We used 1 mmol/L of ZT to treatment the branch by hydroponics and collected flower buds at 0 h, 12 h, 24 h, and 48 h, respectively. 0 h treatment was selected as the control. Total RNA was isolated from flower buds using Trizol reagent (Life Technologies, Carlsbad, CA, USA) followed the manufacturer’s protocols. *AP2* gene expression was confirmed using qRT-PCR.

### Real-time quantitative RT-PCR analysis

Candidate genes and lncRNAs expression were confirmed using qRT-PCR. The same RNA samples were used for the qRT-PCR assays as well as for the RNA-seq experiments. Furthermore, we extracted RNA and miRNA used to detect the expression. Total RNA was used to synthesize the first-strand cDNA using Superscript II reverse transcriptase (Invitrogen, San Diego, CA). Gene-specific primers were designed rendering to gene sequences using software Beacon Designer 7 (Premier Biosoft, Palo Alto CA), showed in Additional file 11. Primers specific for house keeping gene (*RP II*) used to standardize the reactions [64]. qRT-PCR was performed using the ABI 7300 Real-Time PCR System (Applied Biosystems, Foster City, CA, USA) and SYBR Green Real-time PCR Master Mix (Toyobo, Osaka, Japan). Experiments were performed using the method described above [65]. Three technical replicates performed for each biological replicate. The relative expression level of genes and lncRNAs were calculated using 2^-△△CT^ method.

## Additional files

Additional file 1: Statistical data of the mapped reads. (XLS 11 kb)

Additional file 2: The unique reads mapped to various chromosomes (a, b) and the biological replicates correlation coefficient of the samples (c). (JPG 1138 kb)

Additional file 3: The novel lncRNAs predicted in this study. (XLS 105 kb)

Additional file 4: The differential expression genes identified in *Prunus mume* buds by RNA-Seq. (XLS 391 kb)

Additional file 5: Gene ontology (a) and pathway-enrichment (b) analysis for DEGs. (JPG 695 kb)

Additional file 6: Pathway-enrichment analysis for DEGs. (XLS 68 kb)

Additional file 7: The differential expressed lncRNAs in this study. (XLS 63 kb)

Additional file 8: The precursor analysis of the new lncRNAs. (XLS 23 kb)

Additional file 9: Target miRNAs of the DEKLs and DENLs. (XLS 46 kb)

Additional file 10: Differential expression lncRNAs *cis*- and *trans*-target genes. (XLS 100 kb)

Additional file 11: Primers used in real-time quantitative RT-PCR. (DOC 108 kb)

## Abbreviations

ceRNAs: competing endogenous RNAs
CNCI: Coding-Non-Coding Index
CPC: Coding Potential Calculator
DEGs: differentially expressed genes
DEKLs: differentially expressed known lncRNAs
DNELs: differentially expressed novel lncRNAs
FDR: false discovery rate
KEGG: Kyoto encyclopedia of genes and genomes
LncRNAs: long non-coding RNAs
RPKM: Reads per kilobase of transcript per million fragments mapped
SEM: Scanning electron microscopy

## References

[1] Katsarou K, Rao ALN, Tsagris M, Kalantidis K (2015). Infectious long non-coding RNAs. Biochimie.

[2] Li H, Heng D, Dong Z, Ming L, Yanhong L, Fang Z (2018). Systematic identification of long non-coding RNAs during pollen development and fertilization in *Brassica rapa*. Plant J.

[3] Wang M, Yuan D, Tu L, Gao W, He Y, Hu H (2015). Long noncoding RNAs and their proposed functions in fibre development of cotton (*Gossypium* spp.). New Phytol.

[4] Nie L, Wu HJ, Hsu JM, Chang SS, Labaff AM, Li CW (2012). Long non-coding RNAs: versatile master regulators of gene expression and crucial players in cancer. Am J Transl Res.

[5] Liu D, Mewalal R, Hu R, Tuskan GA, Yang X (2017). New technologies accelerate the exploration of non-coding RNAs in horticultural plants. Hortic Res.

[6] Amor BB, Wirth S, Merchan F, Laporte P, D’Aubentoncarafa Y, Hirsch J (2009). Novel long non-protein coding RNAs involved in *Arabidopsis* differentiation and stress responses. Genome Res.

[7] Zhu M, Zhang M, Xing L, Li W, Jiang H, Wang L (2017). Transcriptomic analysis of long non-coding RNAs and coding genes uncovers a complex regulatory network that is involved in maize seed development. Genes..

[8] Wang M, Zhao W, Gao L, Zhao L (2018). Genome-wide profiling of long non-coding RNAs from tomato and a comparison with mRNAs associated with the regulation of fruit ripening. BMC Plant Biol.

[9] Li S, Yu X, Lei N, Cheng Z, Zhao P, He Y (2017). Genome-wide identification and functional prediction of cold and/or drought-responsive lncRNAs in cassava. Sci Rep.

[10] Liu F, Marquardt S, Lister C, Swiezewski S, Dean C (2010). Targeted 3′ processing of antisense transcripts triggers *Arabidopsis FLC* chromatin silencing. Science.

[11] Zhu QH, Wang MB (2012). Molecular functions of long non-coding RNAs in plants. Genes.

[12] Kang C, Liu Z (2015). Global identification and analysis of long non-coding RNAs in diploid strawberry *Fragaria vesca* during flower and fruit development. BMC Genomics.

[13] Swiezewski S, Liu F, Magusin A, Dean C (2009). Cold-induced silencing by long antisense transcripts of an *Arabidopsis* Polycomb target. Nature.

[14] Heo JB, Sung S (2011). Vernalization-mediated epigenetic silencing by a long intronic noncoding RNA. Science.

[15] Li R, Fu D, Zhu B, Luo Y, Zhu H (2018). CRISPR/Cas9-mediated mutagenesis of *lncRNA1459* alters tomato fruit ripening. Plant J.

[16] Paraskevopoulou MD, Hatzigeorgiou AG (2016). Analyzing miRNA-lncRNA interactions. Method Mol Biol.

[17] Wu HJ, Wang XJ (2013). Widespread long noncoding RNAs as endogenous target mimics for microRNAs in plants. Plant Physiol.

[18] Meng, Wang, Hua-Jun, Fang, Chengcai, Xiu-Jie. A long noncoding RNA involved in rice reproductive development by negatively regulating Osa-miR160. Sci Bull 2017;62(7):470–475. 10.1016/j.scib.2017.03.013 36659255

[19] Tian J, Song Y, Du Q, Yang X, Ci D, Chen J (2016). Population genomic analysis of gibberellin-responsive long non-coding RNAs in *Populus*. J Exp Bot.

[20] Werner T, Motyka V, Strnad M, Schmülling T (2001). Regulation of plant growth by cytokinin. P Natl Acad Sci USA..

[21] Young TE, Geisler-Lee J, Gallie DR (2004). Senescence-induced expression of cytokinin reverses pistil abortion during maize flower development. Plant J.

[22] Rashotte AM, Carson SD, Kieber JJ, To JP (2003). Expression profiling of cytokinin action in Arabidopsis. Plant Physiol.

[23] Holt AL, Haperen JMV, Groot EP, Laux T (2014). Signaling in shoot and flower meristems of *Arabidopsis thaliana*. Curr Opin Plant Biol.

[24] Yu H, Ito T, Zhao Y, Peng J, Kumar P, Meyerowitz EM (2004). Floral homeotic genes are targets of gibberellin signaling in flower development. P Natl Acad Sci USA.

[25] Sun L, Yang W, Zhang Q, Cheng T, Pan H, Xu Z (2013). Genome-wide characterization and linkage mapping of simple sequence repeats in mei (*Prunus mume* Sieb. Et Zucc.). PLoS One.

[26] Wang L, Zhao S, Gu C, Zhou Y, Zhou H, Ma J (2013). Deep RNA-Seq uncovers the peach transcriptome landscape. Plant Mol Biol.

[27] Li L, Eichten SR, Shimizu R, Petsch K, Yeh CT, Wu W (2014). Genome-wide discovery and characterization of maize long non-coding RNAs. Genome Biol.

[28] Deng P, Shu L, Nie X, Song W, Liang W (2018). Conservation analysis of long non-coding RNAs in plants. Sci China Life Sci.

[29] Xu Q, Song Z, Zhu C, Tao C, Kang L, Liu W (2017). Systematic comparison of lncRNAs with protein coding mRNAs in population expression and their response to environmental change. BMC Plant Biol.

[30] Guttman M, Rinn JL (2012). Modular regulatory principles of large non-coding RNAs. Nature.

[31] Deng F, Zhang X, Wang W, Yuan R, Shen F (2018). Identification of *Gossypium hirsutum* long non-coding RNAs (lncRNAs) under salt stress. BMC Plant Biol.

[32] Clark SE (1998). Running MP, Meyerowitz EM. *CLAVATA1*, a regulator of meristem and flower development in *Arabidopsis*. Development.

[33] Schoof H, Lenhard M, Haecker A, Mayer KFX, Jürgens G, Laux T (2000). The stem cell population of *Arabidopsis* shoot meristems in maintained by a regulatory loop between the *CLAVATA* and *WUSCHEL* genes. Cell.

[34] Peremyslov VV, Prokhnevsky AI, Dolja VV (2010). Class XI myosins are required for development, cell expansion, and F-actin organization in *Arabidopsis*. Plant Cell.

[35] Xu J, Ding Z, Vizcaybarrena G, Shi J, Liang W, Yuan Z (2014). *ABORTED MICROSPORES* acts as a master regulator of pollen wall formation in *Arabidopsis*. Plant Cell.

[36] Li J, Qin M, Qiao X, Cheng Y, Li X, Zhang H (2017). A new insight into the evolution and functional divergence of SWEET transporters in Chinese white pear (*Pyrus bretschneideri*). Plant Cell Physiol.

[37] Fatica A, Bozzoni I (2014). Long non-coding RNAs: new players in cell differentiation and development. Nat Rev Genet.

[38] Marquardt S, Raitskin O, Wu Z, Liu F, Sun Q, Dean C (2014). Functional consequences of splicing of the antisense transcript COOLAIR on FLC transcription. Mol Cell.

[39] Liu J, Wang H, Chua NH (2015). Long noncoding RNA transcriptome of plants. Plant Biotechnol J.

[40] Suzuki N, Shulaev V, Mittler R (2005). Enhanced tolerance to environmental stress in transgenic plants expressing the transcriptional coactivator multiprotein bridging factor 1c. Plant Physiol.

[41] Krogan NT, Hogan K, Long JA (2012). APETALA2 negatively regulates multiple floral organ identity genes in *Arabidopsis* by recruiting the co-repressor TOPLESS and the histone deacetylase HDA19. Development.

[42] Deyholos MK, Sieburth LE (2000). Separable whorl-specific expression and negative regulation by enhancer elements within the *AGAMOUS* second intron. Plant Cell.

[43] Adamczyk BJ, Fernandez DE (2009). MIKC* MADS domain heterodimers are required for pollen maturation and tube growth in *Arabidopsis*. Plant Physiol.

[44] Ren J, Yang Y, Xue J, Xi Z, Hu L, Pan SJ (2018). Long noncoding RNA SNHG7 promotes the progression and growth of glioblastoma via inhibition of miR-5095. Biochem Bioph Res Co.

[45] Li Z, Kim YJ, Dinh TT, Chen X (2007). miR172 regulates stem cell fate and defines the inner boundary of *APETALA3* and *PISTILLATA* expression domain in *Arabidopsis* floral meristems. Plant J.

[46] Wang T, Pan H, Wang J, Yang W, Cheng T, Zhang Q (2014). Identification and profiling of novel and conserved microRNAs during the flower opening process in *Prunus mume* via deep sequencing. Mol Gen Genomics.

[47] Chen X (2004). A microRNA as a translational repressor of *APETALA2* in *Arabidopsis* flower development. Science.

[48] Chen X, Liu J, Cheng Y, Jia D. *HEN1* functions pleiotropically in *Arabidopsis* development and acts in C function in the flower. Development 2002;129(5):1085. 10.1242/dev.129.5.1085 PMC5137379 11874905

[49] Cartolano M, Castillo R, Efremova N, Kuckenberg M, Zethof J, Gerats T (2007). A conserved microRNA module exerts homeotic control over *Petunia hybrida* and *Antirrhinum majus* floral organ identity. Nat Genet.

[50] Wang W, Shi T, Ni X, Xu Y, Qu S, Gao Z. The role of miR319a and its target gene *TCP4* in the regulation of pistil development in *Prunus mume*. Genome. 2017;61(1). 10.1139/gen-2017-0118 29035682

[51] Liang G, He H, Li Y, Wang F, Yu D (2014). Molecular mechanism of microRNA396 mediating pistil development in *Arabidopsis*. Plant Physiol.

[52] Werner T, Motyka V, Laucou V, Smets R, Onckelen HV, Schmülling T (2003). Cytokinin-deficient transgenic Arabidopsis plants show multiple developmental alterations indicating opposite functions of cytokinins in the regulation of shoot and root meristem activity. Plant Cell.

[53] Deruère J, Maxwell BB, Morris VF, Hutchison CE, Ferreira FJ, To JP (2007). Cytokinin regulates type-a Arabidopsis response regulator activity and protein stability via two-component phosphorelay. Plant Cell.

[54] Liu Z, Li J, Wang L, Li Q, Lu Q, Yu Y (2016). Repression of callus initiation by the miRNA-directed interaction of auxin-cytokinin in *Arabidopsis thaliana*. Plant J.

[55] Cheng Y, Dai X, Zhao Y (2006). Auxin biosynthesis by the YUCCA flavin monooxygenases controls the formation of floral organs and vascular tissues in *Arabidopsis*. Genes Dev.

[56] Wang JW, Wang LJ, Xue HW, Chen XY (2005). Control of root cap formation by MicroRNA-targeted auxin response factors in Arabidopsis. Plant Cell.

[57] Liu T, Wu P, Wang Q, Wang W, Zhang C, Sun F (2018). Comparative transcriptome discovery and elucidation of the mechanism of long noncoding RNAs during vernalization in *Brassica rapa*. Plant Growth Regul.

[58] Shi T, Zhang QL, Gao ZH, Zhen Z, Zhuang WB (2011). Analyses on pistil differentiation process and related biochemical indexes of two cultivars of *Prunus mume*. J Plant Resour Env.

[59] Trapnell C, Pachter L, Salzberg SL (2009). TopHat: discovering splice junctions with RNA-Seq. Bioinformatics.

[60] Nawrocki EP, Burge SW, Bateman A, Daub J, Eberhardt RY, Eddy SR (2015). Rfam 12.0: updates to the RNA families database. Nucleic Acids Res.

[61] Audic S, Claverie JM (1997). The significance of digital gene expression profiles. Genome Res.

[62] Wang XW, Xiong AS, Yao QH, Zhang Z, Qiao YS (2010). Direct isolation of high-quality low molecular weight RNA of pear peel from the extraction mixture containing nucleic acid. Mol Biotechnol.

[63] Ping CY, Yu YW (2005). Determination of GA_3_, IAA, ABA and ZT in dormant buds of *Allium ovalifolium* by HPLC. J Sichuan Agr Univ.

[64] Tong ZG, Gao ZH, Wang F, Zhou J, Zhang Z (2009). Selection of reliable reference genes for gene expression studies in peach using real-time PCR. BMC Mol Biol.

[65] Wu X, Gong Q, Ni X, Zhou Y, Gao Z (2017). UFGT: the key enzyme associated with the petals variegation in Japanese apricot. Front Plant Sci.

