# Supplementary figures and images for: Genome-wide discovery and characterization of flower development related long non-coding RNAs in Prunus mume
Source: BMC Plant Biol. 2019 Feb 11;19:64. doi: 10.1186/s12870-019-1672-7 (PMC6371585; doi:10.1186/s12870-019-1672-7)

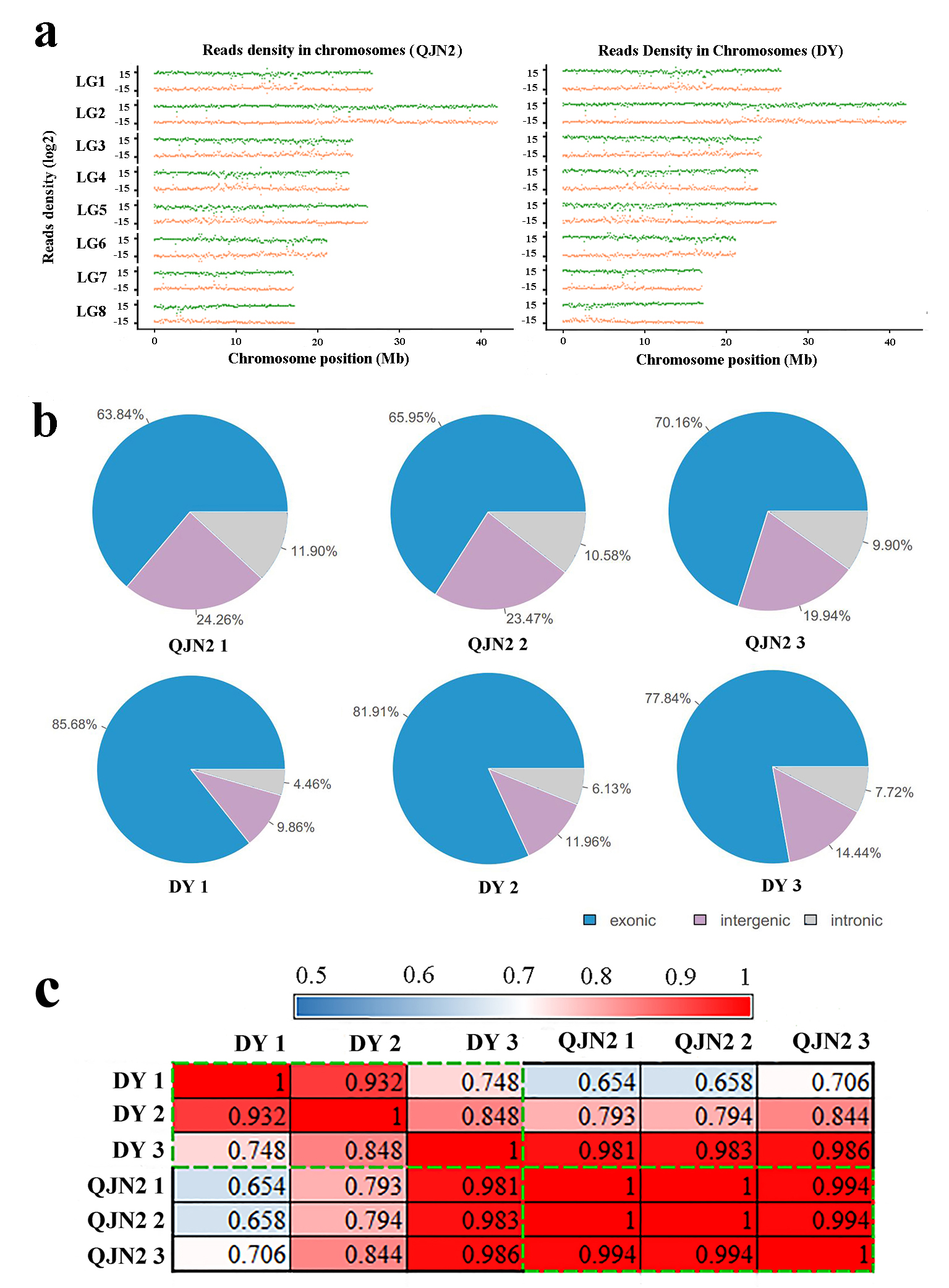

Supplement: Supplementary file 2 — The unique reads mapped to various chromosomes (a, b) and the biological replicates correlation coefficient of the samples (c). (JPG 1138 kb) [file 12870_2019_1672_MOESM2_ESM.jpg]

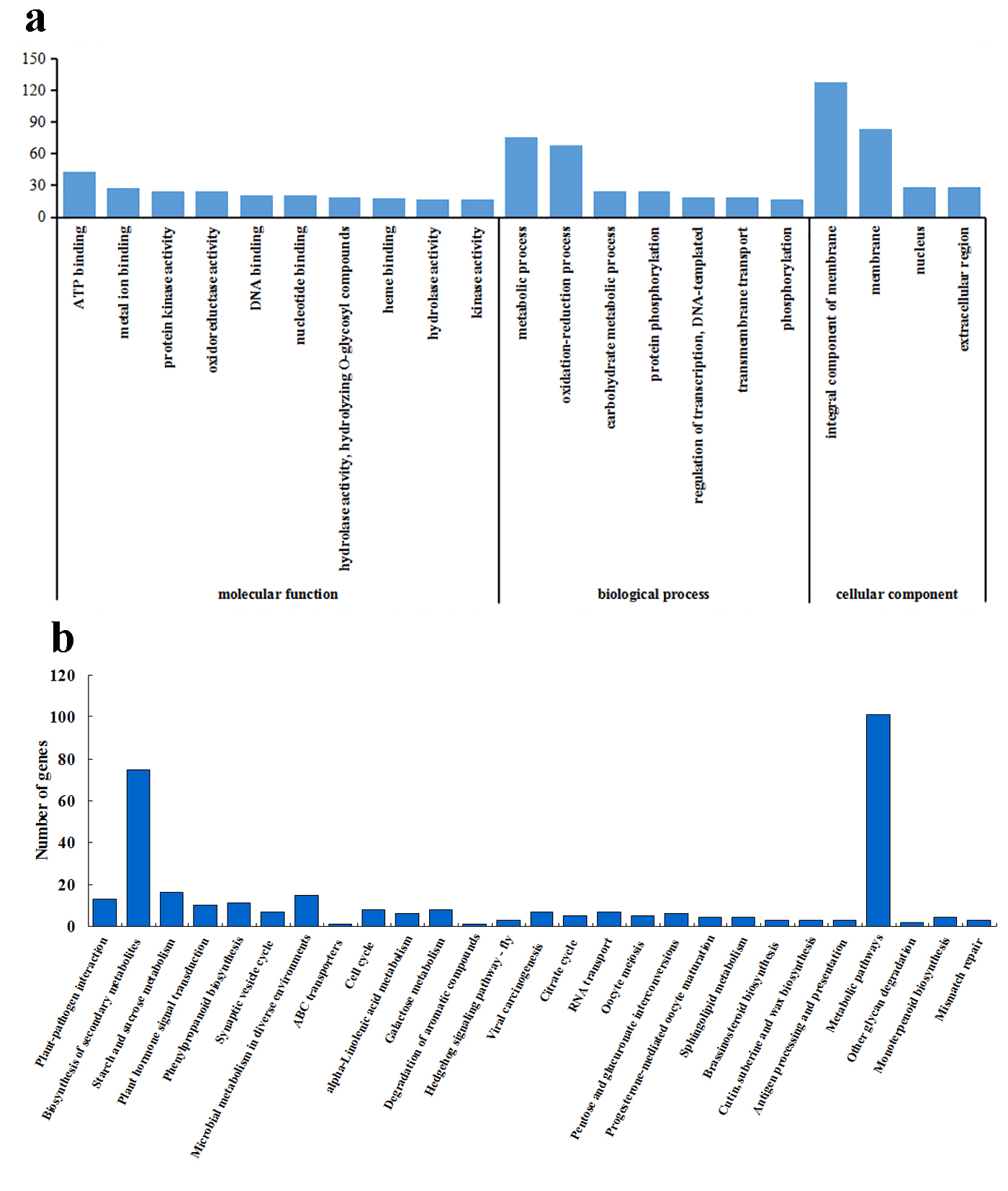

Supplement: Supplementary file 5 — Gene ontology (a) and pathway-enrichment (b) analysis for DEGs. (JPG 695 kb) [file 12870_2019_1672_MOESM5_ESM.jpg]
