# Supplementary material for: Genome-wide discovery and characterization of flower development related long non-coding RNAs in Prunus mume
Source: BMC Plant Biol. 2019 Feb 11;19:64. doi: 10.1186/s12870-019-1672-7 (PMC6371585; doi:10.1186/s12870-019-1672-7)
Supplement: Supplementary file 11 — Primers used in real-time quantitative RT-PCR. (DOC 108 kb) [file 12870_2019_1672_MOESM11_ESM.doc]

Additional file 11 Primers used in real-time quantitative RT-PCR

| *Gene* | *Primer* | **Sequence (5’-3’)** |
| --- | --- | --- |
| XR_513404.2 | Forward | GTCTAGGAAATGCCAAAGG |
|  | Reverse | TGCTCAATCTGGATAAGTCT |
| XR_513468.1 | Forward | CGTTGAATGAGCCATATACC |
|  | Reverse | ACACAAGCGTAAGTCCTAT |
| XR_514079.2 | Forward | GAAGCATTAACCAGCAGAT |
|  | Reverse | CCACAATCAGATAGCCATAC |
| XR_514340.2 | Forward | ACAGCAGGATTGGTAGTT |
|  | Reverse | GCCCTTCTCTTCTTCATTAC |
| XR_514847.2 | Forward | GAGTGGTGGGATATGCTT |
|  | Reverse | GTCTGTATAACTGGCTTGC |
| XR_001677334.1 | Forward | CGGATGTTGGTGGAAATG |
|  | Reverse | CGATAGTGATTGCGTTCTG |
| XR_001677337.1 | Forward | TGTGTCCTTGTTGTGTAGA |
|  | Reverse | TTGTCTTGCCTTACTCAGT |
| XR_001677430.1 | Forward | ATGGCTGACTGTGATGTT |
|  | Reverse | CTCCTCGGACTCGTAATC |
| XR_001677672.1 | Forward | TGAGTTCTTCTGCTTCCAT |
|  | Reverse | CATTCTTCTGTCCTCATTCTC |
| XR_001678033.1 | Forward | CAGGTGTGAAGAGGATGA |
|  | Reverse | TGAGAGTGTGGCAATAGAA |
| XR_001678112.1 | Forward | GCACTATTCATCAGCCAAG |
|  | Reverse | TCGTAACTTCCAACATCCA |
| XR_001678569.1 | Forward | CTGACTGAGAGGCTTAGG |
|  | Reverse | GCATGGAAGGACTGGATA |
| NM_001293267.1 | Forward | GTGATATTGTGGCACTTGAT |
|  | Reverse | TCGGAGCATTCATCTTCA |
| XM_016791880.1 | Forward | CTCCTCTCACTTCTCTTCTT |
|  | Reverse | GCTTGCTTGCCTTGTATT |
| XM_008225845.1 | Forward | TGCTTGGACTCAGAACTT |
|  | Reverse | TGCCTACCCTTTCATTCATA |
| XM_008231094.1 | Forward | ATGTGGATGTTGATGGAGA |
|  | Reverse | GATGAGGAACTTGGAGGAT |
| XM_016794461.1 | Forward | GCTCTACAACTTGCCAATAG |
|  | Reverse | GTGTCAATGTGTCCATAAGG |
| XM_008235879.1 | Forward | TAGGCAGGAGTCTTACAATC |
|  | Reverse | CAATGAGGAGGAGGAAGG |
| XM_008243464.1 | Forward | CTTCTGATAAGGCTTCTGATG |
|  | Reverse | ATAGGCATTGGCAGTTCT |
| XM_008246029.2 | Forward | GGAGAGGAAGGATTGAGATTA |
|  | Reverse | ATGCTGGTGCTTGAGAAT |
| XM_008221784.1 | Forward | TATGATTGCCGAACAGTCT |
|  | Reverse | ATGAGCCACCTTCTTGAA |
| XM_016796611.1 | Forward | TCTTCAGGACTTCTGTGTAG |
|  | Reverse | CTATGCGAGCCAAGGATA |
| XM_008247827.1 | Forward | GTTGTTGGTGTATGTGATGA |
|  | Reverse | TTGGATGTGTAGGAAGTGAT |
| XR_514690.2 | Forward | AGGCTGACCATATTCTTGT |
|  | Reverse | ACACTGCTACCTTCATAACA |
| XR_514769.2 | Forward | GCCAAGAATGCGTTAGAA |
|  | Reverse | CCAACTCATCAACCTTAGC |
| XR_513949.2 | Forward | CGCTGGTAGAATATGAACTG |
|  | Reverse | TACGACTAGACTCCGATAGA |
| XR_001677969.1 | Forward | GCACATTCTCAGTCTTCAC |
|  | Reverse | ACATCATCAGCAGCACTA |
| XR_001678028.1 | Forward | GGAACATTGGCTCAGTATTAG |
|  | Reverse | AGGTAATCGTTGCTCTGT |
| XR_001678297.1 | Forward | GCCTCAACACTTCTATTACTAC |
|  | Reverse | ACCACTCATTCCACCATT |
| TCONS_00032517 | Forward | CCTCATCTATGTAGTTGGAATC |
|  | Reverse | CCGAATTAGTCTGCTTTGA |
| AP2 | Forward | GAGTGGAGGCTGACATAA |
|  | Reverse | AGCATCAATCTCGGTATCA |
| A-ARR | Forward | CAGGAATGACAGGCTATGA |
|  | Reverse | TCTTGTTGATGTTGGATTGG |
| RPⅡ | Forward | TGAAGCATACACCTATGATGATGAAG |
|  | Reverse | CTTTGACAGCACCAGTAGATTCC |
| ppe-miR172d | RT | GTCACATCGTATCGTGAAGCTGCGCAGCTGATGTGACCTGCAGCA |
|  | Forward | TGCACTAGCGTGGGAATCTTGA |
|  | Reverse | ACATCGTATCGTGAAGCTGC |
| ppe-miR396b | RT | GTCACATCGTATCGTGAAGCTGCGCAGCTGATGTGACAAGTTCAA |
|  | Forward | TGCACTAGCGTGTTCCACAGCT |
|  | Reverse | ACATCGTATCGTGAAGCTGC |
| ppe-miR319a | RT | GTCACATCGTATCGTGAAGCTGCGCAGCTGATGTGACGGGAGCTC |
|  | Forward | TGCACTAGCGTGTTGGACTGAA |
|  | Reverse | ACATCGTATCGTGAAGCTGC |
| ppe-miR319b | RT | GTCACATCGTATCGTGAAGCTGCGCAGCTGATGTGACTGGATGAA |
|  | Forward | TGCACTAGCGTGTAGCTGCCGA |
|  | Reverse | ACATCGTATCGTGAAGCTGC |
| ppe-miR169a/b/c | RT | GTCACATCGTATCGTGAAGCTGCGCAGCTGATGTGACCCGGCAAG |
|  | Forward | TGCACTAGCGTGCAGCCAAGGA |
|  | Reverse | ACATCGTATCGTGAAGCTGC |
| ppe-miR169e-5p | RT | GTCACATCGTATCGTGAAGCTGCGCAGCTGATGTGACTGGCAAGT |
|  | Forward | TGCACTAGCGTGTGAGCCAAGG |
|  | Reverse | ACATCGTATCGTGAAGCTGC |
| ppe-miR160a/b | RT | GTCACATCGTATCGTGAAGCTGCGCAGCTGATGTGACTGGCATAC |
|  | Forward | TGCACTAGCGTGTGCCTG |
|  | Reverse | ACATCGTATCGTGAAGCTGC |
| 5S | RT | GTCACATCGTATCGTGAAGCTGCGCAGCTGATGTGACTGGATTGG |
|  | Forward | TGCACTAGCGTGTAGAGGAACC |
|  | Reverse | ACATCGTATCGTGAAGCTGC |
